# Host–Guest Interactions Enhance Charge Transport across Single Cyclodextrin/Azobenzene Complex Junction

**DOI:** 10.1021/jacs.5c20902

**Published:** 2026-03-12

**Authors:** Song Han, Jingjing Zhao, Sumit Naskar, Di Wu, Carmen Herrmann, Jianlong Xia, Haixing Li

**Affiliations:** † Department of Physics, 53025 City University of Hong Kong, Kowloon, Hong Kong 999077, China; ‡ State Key Laboratory of Advanced Technology for Materials Synthesis and Processing, Center of Smart Materials and Devices, and School of Chemistry, Chemical Engineering and Life Science, 12565 Wuhan University of Technology, Wuhan 430070, China; § Department of Chemistry, University of Hamburg, Harbor Bldg. 610, Luruper Chaussee 149, 22761 Hamburg, Germany; ∥ The Hamburg Centre for Ultrafast Imaging, University of Hamburg, Luruper Chaussee 149, 22761 Hamburg, Germany; ⊥ International School of Materials Science and Engineering, 12565 Wuhan University of Technology, Wuhan 430070, China

## Abstract

While azobenzene has been studied extensively for its single-molecule charge transport properties, its complexation with guest ring molecules may significantly influence charge transport that is not yet well understood. In this work, we study the influence of host–guest interactions between α-cyclodextrin (α-CD) and azobenzene on the single-molecule conductance of azobenzene in an aqueous solution. Hydrophobicity of azobenzene drives its formation of an α-CD/azobenzene host–guest complex with α-CD in water, which is indicated in our nuclear magnetic resonance and ultraviolet–visible spectroscopy experiments. We see a modest ∼3.5-fold conductance increase for amine-terminated azobenzene upon host–guest complex formation. Notably, this enhancement displays progressive conductance attenuation over time, finally down to the conductance value of the azobenzene junction, which we attribute to the declining number of formed complexes in the aqueous solution as α-CD aggregates with time. In contrast, for amine-terminated stilbene (backbone modification) and for thiomethyl-terminated azobenzene (linker modification), no conductance change is seen with the addition of α-CD. First-principles simulations suggest that the lowest unoccupied molecular orbital (LUMO) of the α-CD/amine-azobenzene complex junction is at a lower energy than that of amine-azobenzene, thereby suggesting a possible conductance increase, agreeing with our experimental observations. Taken together, this study provides valuable perspectives on the intricate roles that the host–guest interactions play in regulating the molecular electronic properties.

## Graphical abstract

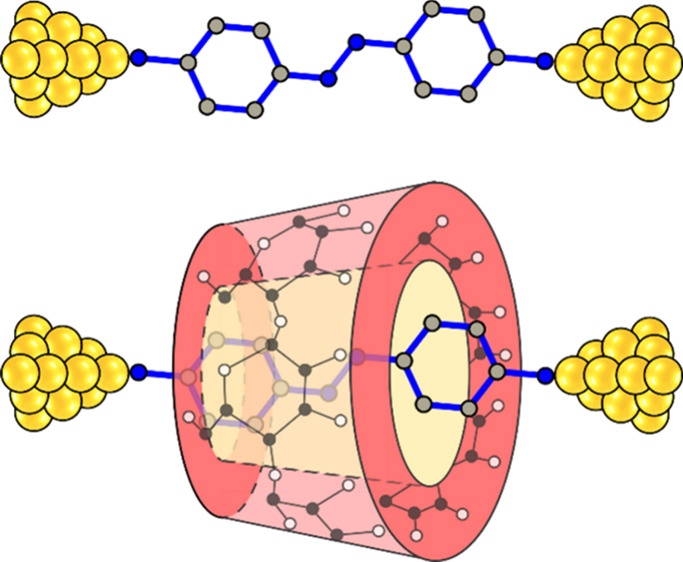

## Introduction

In the realm of molecular electronics, supramolecular electronics is an emerging branch that utilizes noncovalent interactions—such as hydrogen bonding,^1–4^ π–π stacking, metal-coordination bonding, and host–guest recognition—to construct functional molecular-scale electronic devices. Unlike conventional electronics based on rigid covalent structures, supramolecular systems exhibit dynamic, flexible, and stimuli-responsive properties, enabling the development of smart electronic materials with self-healing, reconfigurable, and environmentally adaptive capabilities.^4–9^ These advantages make supramolecular electronics particularly promising for applications in flexible electronics, biosensing, and neuromorphic computing.^10–13^

Host–guest chemistry offers a unique handle for controlling and modulating charge transport in molecular-scale devices.^14–16^ Cucurbituril (CB) is one of the most widely studied host molecules in forming single host–guest complex junctions due to its two important properties.^17–20^ First, CB has recognition of selective guest molecules, particularly for hydrophobic and cationic ones. Second, for those that can form host–guest complexes with CB host, CB generally has a high binding affinity with an association constant *K*_a_ that can be as high as 10^15^ M^–1^.^21^ Both of these characteristics of CB enable it to form stable single-complex junctions. Zhang and co-workers demonstrated that viologen encapsulation within the hydrophobic Cucurbit[8]­uril (CB[8]) cavity leads to a 3.4-fold enhancement in molecular conductance of viologen, arising primarily from modulation of outer-sphere reorganization energy upon host–guest complexation.^9^ In another investigation, through an integrated approach combining scanning tunneling microscope-based break junction (STM-BJ) measurements with nuclear magnetic resonance (NMR) spectroscopy, Yuan and colleagues systematically characterized the CB[8]-mediated dimerization of 1,2-bis­(4-pyridinyl)­ethylene.^22^ Their comparative analysis revealed the superior sensitivity of the STM-BJ technique in monitoring confined host–guest reactions, achieving detection of reaction products at ultralow reactant concentration (5 × 10^–6^ M), where conventional NMR measurements proved ineffective. In a separate study, Xiao and co-workers pioneered a supramolecular recognition tunneling platform employing CB[7]-functionalized gold electrodes.^23^ This system facilitates dynamic analyte capture through selective host–guest interactions, generating distinct single-molecule conductance signatures. Through this approach, the authors identified structurally analogous pharmaceuticals and their mixtures, with selectivity tuned through pH modulation and the use of ions such as Na^+^ and Ca^2+^. Although we see many successful examples of using cucurbiturils as host molecules in creating single-molecule junctions,^24^ other macrocycles such as cyclodextrins and crown ethers^25–27^ have still been rarely seen in supramolecular electronic devices despite their wide-ranging applications in other fields.^28–35^

Azobenzene derivatives have been shown as versatile guest molecules in forming diverse host–guest complexes that have been applied in drug delivery, molecular switches, optoelectronic devices, and adaptive materials, due to their unique photoswitching behavior, excellent biocompatibility, multistimuli responsiveness, and tunable molecular architecture.^36–38^ Tan and co-workers systematically investigated the conductance of azobenzene-based single-molecule junctions with varying terminal anchoring groups.^39^ Their studies revealed a counterintuitive phenomenon: while UV irradiation induced the expected trans-to-cis photoisomerization of azobenzene backbones, the resulting conductance changes exhibited anchor-dependent trends. Specifically, junctions with electron-rich amine anchoring groups showed increased conductance upon trans-to-cis isomerization, whereas those with electron-deficient pyridyl anchoring groups displayed decreased conductance. In the same year, Wu and colleagues designed and synthesized azobenzene derivatives with only one thiomethyl terminal linker on one end for creating a light-controlled molecular switch.^40^ Although single-molecule conductance of azobenzene has been investigated extensively, in particular regarding its photoswitching behavior under UV and visible light,^39,41–44^ we have not found many cases where azobenzene is used as the guest molecule in forming host–guest supramolecular junctions.

Single-molecule junctions are often linked by dative or covalent interactions between terminal groups on the organic compounds and Au electrodes,^45^ and the electron tunneling through the molecular backbone, for example, in junctions of alkanes and polyphenylenes, shows an exponential decay with increasing molecular length.^46^ Although such chemical structures are considerably shorter and simpler in comparison to nucleic acids and proteins,^47–51^ supramolecular interactions, such as hydrogen bonding occur in both organic molecules^52^ and biological systems,^53^ and such mechanistic understanding of the roles that supramolecular interactions play in controlling the charge transfer is important to both fields.

In this work, we investigate the impact of host–guest interactions on single-molecule conductance using a model system comprising α-cyclodextrin (α-CD) and azobenzene—a widely studied supramolecular pair.^54–56^ Our experiments of amine-terminated azobenzene reveal that host–guest complexation induces an approximate 3.5-fold enhancement in molecular conductance. Although this conductance increase for the host–guest complex in comparison to the guest junction is modest, it is a robust conductance change that is consistently seen in repeated experiments. In contrast, the conductance of thiomethyl-terminated azobenzene remains the same upon host–guest complex formation. The observed conductance increase for the α-CD/amine-terminated azobenzene host–guest complex is attributed to a closer alignment between the lowest unoccupied molecular orbital (LUMO) transmission peak and the Fermi level, as suggested by our computational results.

## Results and Discussion

We first study the electron transport properties of guest molecule **Azo-NH**_**2**_ (Figure 1a) and host–guest complex formed by guest molecule **Azo-NH**_**2**_ bound within the host ring α-cyclodextrin (α-CD) (Figure 1b). We apply a scanning tunneling microscope-based break-junction (STM-BJ) technique under ambient conditions for measuring the single-molecule junction conductance (Supporting Information Section I and Tables S1 and S2 for details).^59,60^ Schematics illustrating the single-molecule and single-complex junctions are given in Figure 1c. In detail, we prepared a solution of 0.5 mM **Azo-NH**_**2**_ in the commonly used nonpolar solvent 1,2,4-trichlorobenzene (TCB) for conductance experiments. As **Azo-NH**_**2**_ has a low solubility in water and its hydrophobicity drives it to move inside the host ring of α-CD to form host–guest complexes,^61,62^ we use H_2_O as the solvent to facilitate the complex formation. We prepare a solution of 0.2 mM **Azo-NH**_**2**_ and 8 mM α-CD in H_2_O for conductance experiments; in order to exclude any solvent effect on the single-molecule charge transport properties, we also perform conductance measurements for 1 mM **Azo-NH**_**2**_ in H_2_O. We note that when H_2_O was used as the solvent, due to its high dielectric constant, we performed the experiments with Au tips that were insulated by Apiezon wax in order to reduce the background current.^63,64^ We observe a single-molecule conductance of ∼1.0 × 10^–3^ *G*_0_ for **Azo-NH**_**2**_ measured in either TCB or H_2_O (Figures 1d,e and S2), in agreement with previous reports.^39^ We additionally note that a measurement of **Azo-NH**_**2**_ in H_2_O solution upon evaporation of the solvent (i.e., dry measurement) with a regular noncoated tip also reveals the same ∼1.0 × 10^–3^ *G*_0_ conductance peak value (Figure S3).

**Figure 1:**
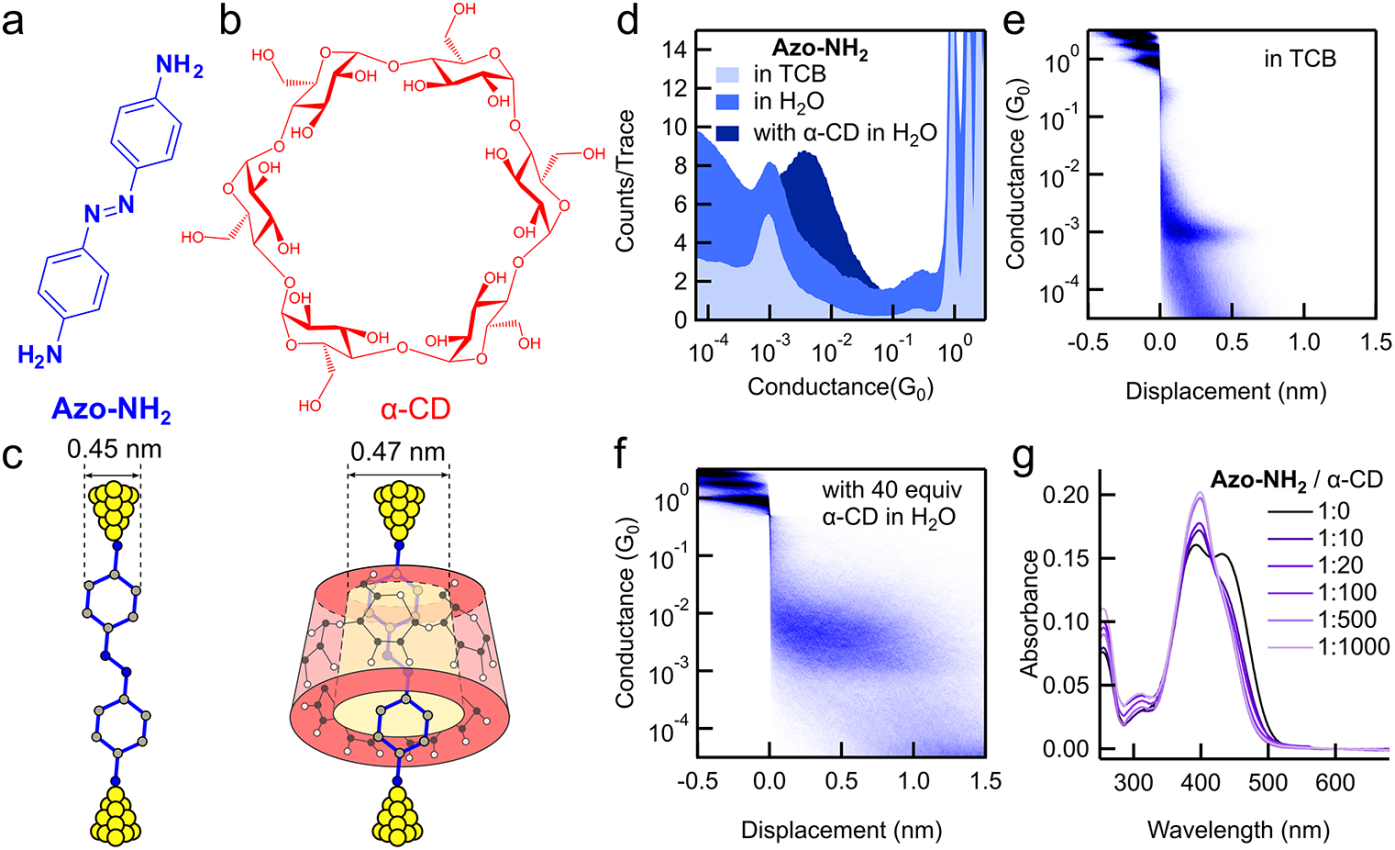
(a, b) Chemical structures of (a) guest molecule **Azo-NH**_**2**_ and (b) host molecule α-cyclodextrin (α-CD). (c) Schematic representation of molecular junctions of the guest molecule (left) and host–guest complex (right) formed in STM-BJ experiments. The sizes for guest molecule^57^ and for host cavity diameter^58^ are indicated. (d) 1D conductance histograms of **Azo-NH**_**2**_ measured in TCB (0.5 mM), in H_2_O (1 mM), and in H_2_O (0.2 mM) with the addition of 40 equiv of α-CD. When H_2_O is the solvent, a wax-coated tip is used. (e, f) 2D conductance histograms for **Azo-NH**_**2**_ measured (e) in TCB (0.5 mM) and (f) in H_2_O (0.2 mM) with the addition of 40 equiv of α-CD. (g) UV–vis absorption spectra of **Azo-NH**_**2**_ measured in the solvent of H_2_O upon the addition of excess α-CD. The concentration of **Azo-NH**_**2**_ is kept at 10 μM, and the concentration ratio between **Azo-NH**_**2**_ and α-CD are indicated in the graph.

In stark contrast, when 40 equiv of α-CD was added to the molecular solution of **Azo-NH**_**2**_, the single-molecule conductance peak value was increased to ∼3.5 × 10^–3^ *G*_0_ (Figure 1d,f). We find that no conductance peaks were observed for a measurement of 40 mM host molecule α-CD (Figure S4), indicating that the conductance peak observed for a mixture of **Azo-NH**_**2**_ and α-CD is not a result of the molecular junctions formed by the host molecule. We attribute this conductance peak, which is higher than the one observed for **Azo-NH**_**2**_ by a factor of 3.5, to be the single-molecule conductance for an α-CD/azobenzene host–guest junction, as illustrated in Figure 1c on the right. To further verify the complexation between **Azo-NH**_**2**_ and α-CD, we carry out UV–vis spectroscopy experiments of **Azo-NH**_**2**_ in the absence and presence of α-CD. As shown in Figure 1g, the absorption peak at 432 nm is significantly suppressed, and the peak at 398 nm is growing in intensity upon the addition of increasing amounts of α-CD. We analyze the peak at 398 nm for determining the association constant next, as it indicates an increased molar extinction coefficient resulting from host–guest complexation. We emphasize that only one guest molecule can fit into one host ring, as suggested by the size of 0.45 nm guest and 0.47 nm host cavity diameter (illustrated in Figure 1c).^57,58^ Then, according to the modified Benesi–Hildebrand equation,^65^ the association constant *K*_Azo‑NH2/α‑CD_ for the 1:1 inclusion complex of α-CD with **Azo-NH**_**2**_ is 4.1 × 10^3^ M^–1^ (Figure S5), suggesting that **Azo-NH**_**2**_ primarily exists in host–guest complex form (×32) in **Azo-NH**_**2**_/α-CD solutions when α-CD is in excess.

Interestingly, we observe that the high single-molecule conductance of the host–guest complex sustains for ∼25 min before a gradual decay until the conductance reaches the low conductance value of the guest junctions under these concentration conditions. In Figure 2a,d, we present 1D and 2D conductance histograms compiled from combined data from four independent experiments of 1 mM **Azo-NH**_**2**_ measured in the presence of 40 mM α-CD in H_2_O with a wax-coated tip (data from each experiment are given in Figures S6–S9). In Figure 2a, the peak corresponding to 25 m indicates that in the initial 25 min of the measurement, the most probable conductance of the molecular junctions is 3.3 × 10^–3^ *G*_0_. As the measurement continues, the most probable conductance gradually decreases, as indicated by the black arrow. This molecular junction conductance drops to 1.9 × 10^–3^ *G*_0_ in 25–50 min, then further declines to 1.3 × 10^–3^ *G*_0_ in 50–75 min, until the conductance stabilizes at ∼1.0 × 10^–3^ *G*_0_ after 75 min.

**Figure 2:**
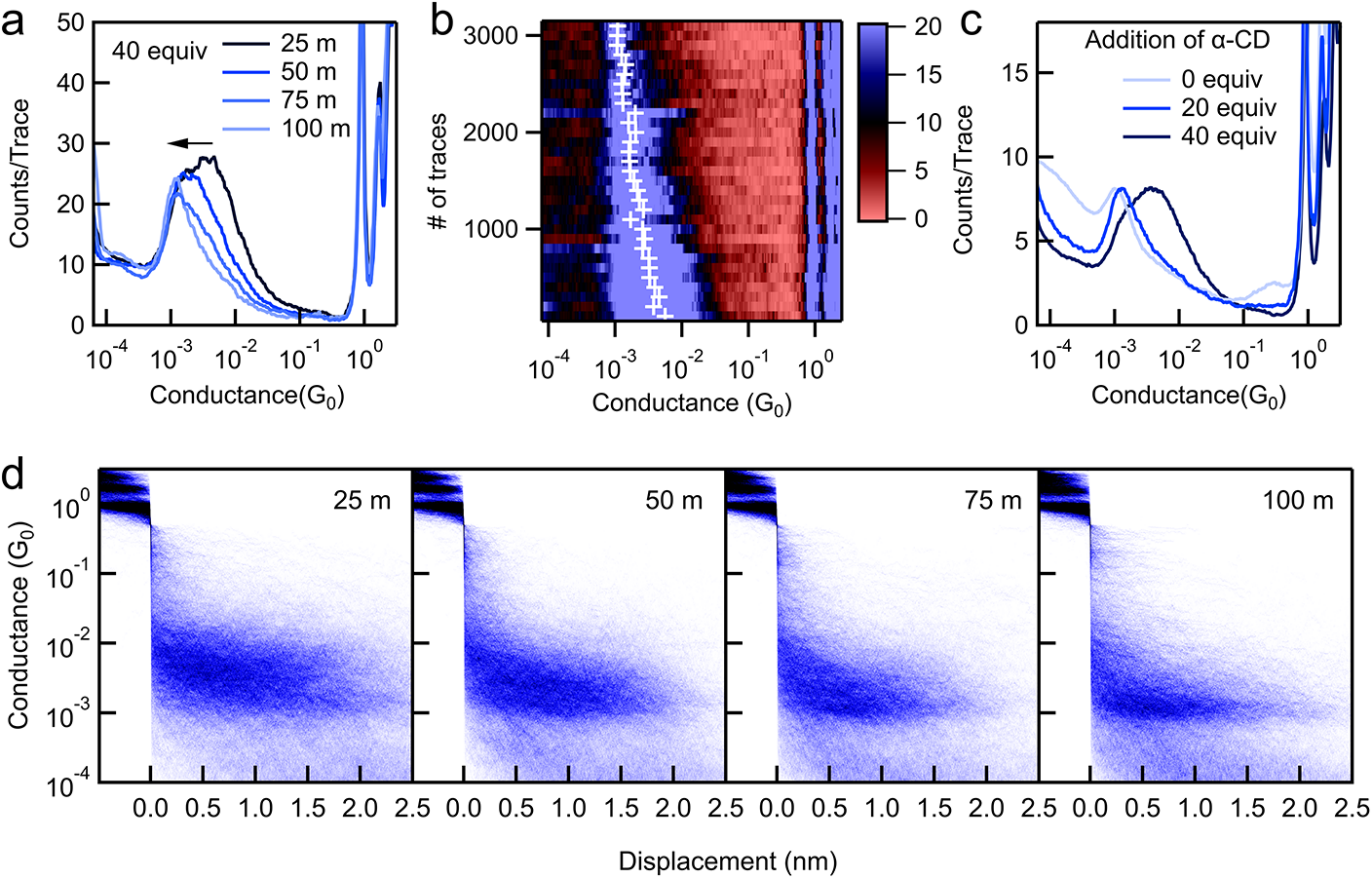
(a) 1D histograms of **Azo-NH**_**2**_ (1 mM) measured in the presence of 40 equiv of α-CD in H_2_O under a bias of 0.1 V in 0–25 min, 25–50 min, 50–75 min, and 75–100 min. For each histogram compiled from traces collected in a ∼25 min time period, experiments were repeated 4 times, and conductance traces collected from 4 independent experiments are combined to generate these histograms. Data for each independent experiment are provided in SI Figures S6–S9. (b) Conductance as a function of time in a 90 min experiment showing a clear transition for the single-molecule conductance peak from a high conductance value (∼6.3 × 10^–3^ *G*_0_) at the start of the measurement to a low one (∼1.0 × 10^–3^ *G*_0_) at the end. The white ‘+’ represents the most probable junction conductance obtained from Gaussian fits to the conductance peaks. Data from three other repeated experiments are given in Figure S10. (c) 1D histograms of **Azo-NH**_**2**_ (1 mM) measured in the absence of α-CD and **Azo-NH**_**2**_ (0.2 mM) measured in the presence of 20 and 40 equiv of α-CD. (d) 2D conductance-displacement histograms compiled from the same data as that used for constructing the 1D histograms in panel (a).

To obtain a quantitative determination of the conductance decay as a function of measurement time for the **Azo-NH**_**2**_/α-CD system, we further plot the conductance distribution against time for the four independent experiments (Figures 2b and S10). Figure 2b illustrates a clear transition in the conductance peak from a high value (∼6.3 × 10^–3^ *G*_0_) at the start of the experiment to a low value (∼1.0 × 10^–3^ *G*_0_) by the 90 min measurement time. Together with the three other repeated experiments (Figure S10), we find that this conductance decay occurs progressively and no high-to-low switching point is identified.

We find in the literature that spontaneous aggregation of α-CD is occurring in aqueous solutions.^66^ Specifically, it has been shown that in an aqueous solution of 10 mM α-CD, the average size of the α-CD aggregates increases with time, and such aggregates could adsorb onto the metal/solution interface through a diffusion mechanism over several hours.^67^ We note that our measurement is accompanied by evaporation of the water solvent, which exacerbates the aggregation of α-CD. Specifically, from a visual inspection, one drop of 40 mM α-CD water solution is fully evaporated in ∼40 min. We thus further perform experiments in a liquid cell, where we do not have complete evaporation of the solvent (Figures S11 and S12), and we continue to see the conductance decay with time, indicating that α-CD aggregation still occurs. We propose that the formation of such aggregates will significantly reduce the amount of α-CD available for forming the host–guest complexes, thereby inhibiting the Au-**Azo-NH**_**2**_/α-CD-Au complex junction formation, leading to the gradual disappearance of the high conductance peak that results from such host–guest complex junctions. By 75 min of the experiment, only free **Azo-NH**_**2**_ molecules remaining in the solution are able to form molecular junctions, giving rise to the low conductance peak at ∼1.0 × 10^–3^ *G*_0_. In addition to this aggregation of the α-CD hypothesis, we also cannot exclude the possibility that the gold surface contributes to the destruction of the host–guest complex.

When we turn to the 2D histograms in Figure 2d, we find that the molecular junction elongation length is the same for the host–guest complex (high conductance ∼3.5 × 10^–3^ *G*_0_) and the guest molecule (low conductance ∼1.0 × 10^–3^ *G*_0_). This observation is in agreement with previous reports of STM-BJ experiments of viologen guest molecules and viologen-CB[8] complexes.^9^ Notably, the junction elongation length for **Azo-NH**_**2**_ is increased slightly when measurements were performed in water with a wax-coated tip, and for host–guest junctions, the junction elongation length is further increased slightly under high host/guest concentrations (0.2 mM in Figure 1f versus 1 mM in Figure 2d); the underlying cause requires further investigation, and we note that similar phenomena have been reported previously.^1,68,69^

Next, the effect of molar ratio between **Azo-NH**_**2**_ and α-CD on the conductance of the **Azo-NH**_**2**_/α-CD system is further investigated in Figures 2c and S13. The addition of 20 equiv of α-CD is insufficient to establish junction formation of **Azo-NH**_**2**_/α-CD host–guest complexes, as the conductance remains similar to that of **Azo-NH**_**2**_ junctions. Further increasing the α-CD concentration to a molar ratio of 1:80 between **Azo-NH**_**2**_ and α-CD does not result in a further conductance increase, which implies that a solution of a molar ratio of 1:40 between **Azo-NH**_**2**_ and α-CD has likely enabled us to obtain a maximized formation of the inclusion complex junctions (Figure S13).

Furthermore, we investigate the conductance of the **Azo-NH**_**2**_/α-CD system in basic aqueous solutions. The results revealed that a basic solvent environment (pH = 12 and 14) disrupts the formation of stable host–guest molecular junctions (Figures S14 and S15). We note that acidic conditions are not systematically studied, as protonation of the amine anchoring group (donation of the lone pair of electrons to the H^+^ ion) would severely compromise their binding affinity to gold electrodes,^70,71^ disabling the formation of host–guest junctions. Overall, we conclude that pH has an impact on the host–guest junction formation, and a neutral solution is preferred for achieving robust complex junction formation.

To further compare the host–guest complex junction with the guest junction, we conduct flicker noise analysis, an approach that provides critical insights into the through-space vs through-bond molecule-electrode coupling schemes.^72^ As established in prior studies, when charge transport across a molecular junction occurs predominantly via through-bond coupling, the normalized noise power (normalized by conductance) exhibits a linear dependence on the conductance. Conversely, a quadratic scaling relationship is characteristic of through-space coupling. We find the value of the scaling exponent (*n*) is 1.05 for **Azo-NH**_**2**_ measured in TCB (Figure 3a), consistent with previously reported values for the same compound.^39^ This observation indicates that charge transport occurs primarily via through-bond pathways in the **Azo-NH**_**2**_ junction.

**Figure 3:**
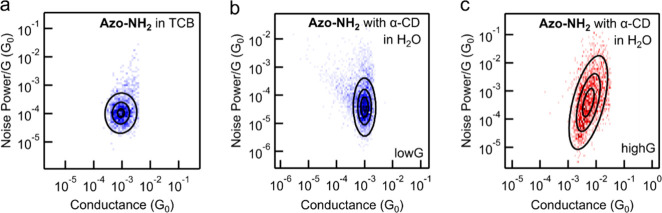
Two-dimensional histogram of normalized flicker noise power against average junction conductance for (a) **Azo-NH**_**2**_ in TCB and (b, c) **Azo-NH**_**2**_ in H_2_O with 40 equiv of α-CD added. We use traces that exhibit an average junction conductance between 2.0 × 10^–4^ and 2.0 × 10^–3^ *G*_0_ in panel (b) low*G* and the ones that exhibit an average junction conductance between 2.0 × 10^–3^ and 6.3 × 10^–2^ *G*_0_ in panel (c) high*G*. Analysis details are given in Supporting Information Section II. The scaling exponents determined for data in panels (a), (b), and (c) are 1.05, 0.98, and 2.36, respectively.

For the **Azo-NH**_**2**_/α-CD host–guest complex, as described above, we observe a gradual decrease in conductance from the high*G* (3.5 × 10^–3^) to the low*G* (1.0 × 10^–3^), which we assign to the **Azo-NH**_**2**_/α-CD and **Azo-NH**_**2**_ junctions, respectively. Thus, we separate the conductance traces that display high*G* and low*G* conductances into two groups and suggest that only the traces that exhibit high*G* arise from the **Azo-NH**_**2**_/α-CD junctions. In Figure 3b,c, we show the 2D histograms of normalized flicker noise power against average junction conductance for high*G* and low*G*. We obtain a scaling exponent value of 0.98 for the low*G*, which closely aligns with the scaling exponent value observed for **Azo-NH**_**2**_ in TCB. This agreement suggests that the uncomplexed **Azo-NH**_**2**_ in polar H_2_O and nonpolar TCB solvent shows the same through-bond charge transport characteristic.

In contrast, the high*G* exhibits a scaling exponent value of 2.36, indicating direct through-space electron injection from the electrodes to the molecule when **Azo-NH**_**2**_ forms a complex with α-CD, which potentially contributes to the overall higher electronic conduction that we see. We hypothesize that the hydroxyl groups at the rim of α-CD are close in distance to both the Au electrodes and the amine anchor of **Azo-NH**_**2**_, which possibly renders a N→Au interaction with less mechanical constraint, i.e., a through-space character. We highlight that this result showcases that through-space transport does not always indicate low conductance.^73,74^ We cannot exclude the possibility that, in addition to or alternatively to electronic effects of α-CD complexation, additional through-space electron injection at the molecule/metal interface might contribute to the increased conductance we observe in the α-CD/**Azo-NH**_**2**_ complex junctions. To ensure reproducibility, two independent sets of flicker noise measurements were performed (Figure S16).

We perform first-principles Kohn–Sham density functional theory (DFT) calculations (details are in the SI) to unravel the nature of charge transport in **Azo-NH**_**2**_ in or without the presence of α-CD. Electron transport is modeled assuming coherent tunneling, described by a nonequilibrium Green’s function (NEGF) approach. Our NEGF-DFT calculations help understand the experimental observation of ∼3.5 times higher conductance in the presence of α-CD. They suggest that the α-CD host modulates the **Azo-NH**_**2**_ wire such that it lowers the energy of the molecular orbitals. In particular, the LUMO in α-CD/**Azo-NH**_**2**_ compared to **Azo-NH**_**2**_ is shifted closer to the Fermi level of the gold (which we estimate to be near −4 eV; data is given in Figure 4), resulting in a larger transmission at the Fermi energy *T*(*E*_F_) and thus a larger zero-bias conductance *G*(0V) = *T*(*E*_F_)*G*_0_.

**Figure 4:**
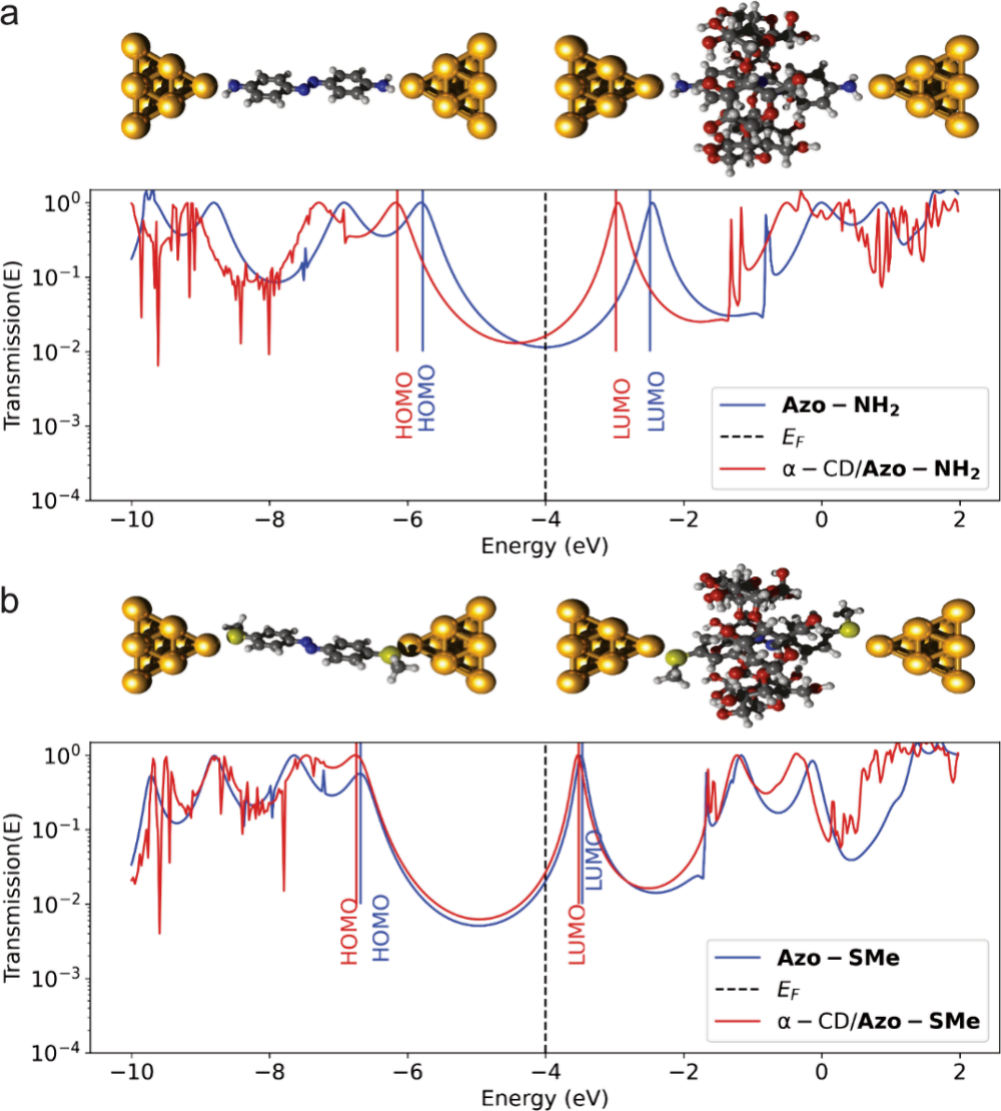
Optimized junction structures and transmission plots for (a) **Azo-NH**_**2**_ and α-CD/**Azo-NH**_**2**_ complex junctions and (b) **Azo-SMe** and α-CD/**Azo-SMe** complex junctions. Solid lines indicate HOMO and LUMO for central subsystem MOs, and black dashed lines indicate an estimated position of the Fermi level *E*_F_ (see the main text for a discussion of the associated uncertainty). The increase in zero-bias conductance (estimated from *T*(*E*_F_)) for **Azo-NH**_**2**_ upon complexation would be estimated as about 1.5 for this choice (with larger values possible for Fermi levels closer to the LUMO peak).

It should be noted though that the exact location of the Fermi level in single-molecule break junctions is difficult to estimate from DFT calculations, for various reasons, such as the challenge of describing both molecular and metallic systems appropriately with one approximate exchange–correlation functional, the difficulty of DFT with describing image charges, and the dependence of the Fermi energy on structural aspects such as the atomistic shape of the electrodes and adsorbed molecules besides the bridging one. It is usually assumed to fall within a window of about −5 to −4 eV, and depending on where exactly it is, the conclusions from our simulations may be different. Yet, we want to point out that our simulations are *consistent* with the rationalization of the conductance increase presented above (although not at the exclusion of other hypotheses). Similarly, we need to take into account that our electrodes are modeled by 10-atom gold clusters. This, together with the approximate nature of our exchange–correlation functional, may affect the reliability of estimated MO level shifts upon complexation. This may be of relevance insofar as the isolated molecules do not show any clear energy changes in the frontier molecular orbitals for the guest system upon host–guest assembly (Figure S17), in contrast to the molecular subsystem MOs in the presence of the gold clusters (Figure S20).

What we have not seen from our molecular structure optimizations are potential hydrogen bonds that could form between host and guest and could potentially provide an alternative hypothesis for the conductance increase. To fully exclude such a hypothesis, however, we would need to do a full statistical sampling of all relevant conformational spaces with or without the solvent, which is currently out of scope for this study due to the large computational effort involved.

We should also note that our computed estimate of about 10^–2^ *G*_0_ for the zero-bias conductance of the **Azo-NH**_**2**_ junction is roughly in line with the typical overestimation of conductance values from DFT-based simulations in the coherent tunneling regime. Along with the chemical structure and length of the molecular wire, this suggests that coherent tunneling is indeed the dominant transport mechanism here. This is in contrast to ref 9, where redox centers were part of the wire, and accordingly, a hopping-like transport mechanism was assumed, leading to a different (reorganization-energy-based) effect of the host on the conductance. While we cannot fully exclude such a mechanism here, our computational results are consistent with an energy shift of MOs upon host–guest complexation within the coherent tunneling regime.

To determine if the N–N double bond in azobenzene is critical for the formation of host–guest complex and the role of N–N double bond in affecting the molecular conductance for the host–guest complex, we next study the charge transport properties of **Sti-NH**_**2**_, a compound of the same structure as **Azo-NH**_**2**_, except that the central N–N double bond is replaced by a C–C double bond (structure in Figure 5a). We first perform STM-BJ experiments of 0.5 mM **Sti-NH**_**2**_ in TCB and in H_2_O, in the absence of α-CD (illustrated in Figure 5b). The 1D conductance histograms reveal that **Sti-NH**_**2**_ exhibits nearly identical conductance values of 7.9 × 10^–4^ and 8.7 × 10^–4^ *G*_0_ in the solvent of TCB and H_2_O, respectively (Figure 5c). When 40 equiv of α-CD were added to a 0.2 mM **Sti-NH**_**2**_ aqueous solution, the molecular conductance peak remains at 9.8 × 10^–4^ *G*_0_. A comparison of the 2D conductance histograms of **Sti-NH**_**2**_ measured in TCB, in H_2_O, and with the addition of α-CD in H_2_O shows that the molecular junction elongation length is not affected by the addition of α-CD (Figures 5d,e and S26). Two possible scenarios can give rise to this phenomenon: **Sti-NH**_**2**_ does not form host–guest complexes in the presence of α-CD, or the **Sti-NH**_**2**_/α-CD complex shows the same conductance as **Sti-NH** **2**. To distinguish these two possibilities, we next perform NMR experiments to determine if **Sti-NH**_**2**_/α-CD host–guest complexes are indeed formed.

**Figure 5:**
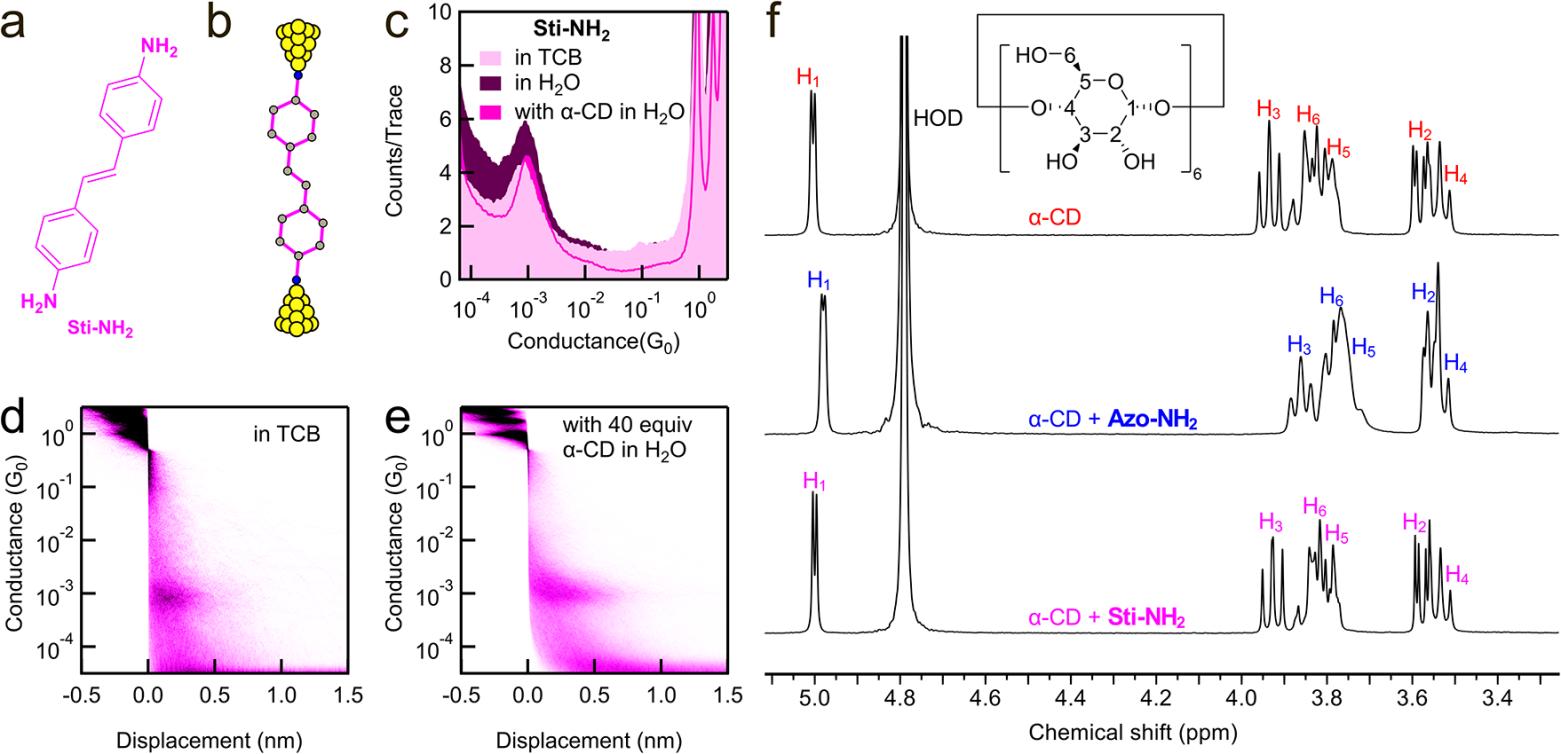
(a) Chemical structure of the guest molecule **Sti-NH**_**2**_. (b) Schematic representation of a single-molecule junction of **Sti-NH**_**2**_. (c) 1D conductance histograms of **Sti-NH**_**2**_ measured in TCB (0.5 mM), in H_2_O (0.5 mM) and in H_2_O (0.2 mM) with the addition of 40 equiv of α-CD. (d, e) 2D conductance histogram for **Sti-NH**_**2**_ measured (d) in TCB and (e) with addition of 40 equiv of α-CD in H_2_O. The proposed corresponding molecular junction measured in panels (d) and (e) are shown in panel (b). (f) ^1^H NMR spectra (400 MHz) for α-CD, a mixture of α-CD and **Azo-NH**_**2**_ (molar ratio of 2:1), and a mixture of α-CD and **Sti-NH**_**2**_ (molar ratio of 2:1) in D_2_O. Inset: chemical structure of α-CD with the proton positions labeled.

In Figure 5f, we present the ^1^H NMR spectra of α-CD, α-CD with **Sti-NH**_**2**_, and α-CD with **Azo-NH**_**2**_. Upon the addition of **Azo-NH**_**2**_, the chemical shifts of protons H_1_, H_3_, H_5_, and H_6_ on α-CD move upfield, suggesting that host–guest interactions are formed between α-CD and **Azo-NH**_**2**_. In contrast, the ^1^H NMR spectrum of α-CD remains unchanged with the addition of **Sti-NH**_**2**_, indicating a possible mere physical mixture between α-CD and **Sti-NH**_**2**_, where no intermolecular interactions between **Sti-NH**_**2**_ and the hydroxyl groups on α-CD are present. We note that in the UV–vis absorption data of **Sti-NH**_**2**_ in the presence of increasing concentration of α-CD, we do see an increase in the intensity of the absorption peak at ∼340 nm for **Sti-NH**_**2**_ (Figure S27), suggesting possible intermolecular interactions between **Sti-NH**_**2**_ and α-CD (the increase in intensity is possibly due to the increased solubility of **Sti-NH**_**2**_ when interactions between **Sti-NH**_**2**_ and α-CD occur^75–77^ ), such as the ones where one amine linker group is enclosed inside the cavity and cannot be attached to the electrodes.^22^ Taken together, based on these observations, we infer that in the STM-BJ experiments of **Sti-NH**_**2**_ in the presence of α-CD, no host–guest complexes are captured between the two electrodes, and the conductance peak corresponds to **Sti-NH**_**2**_ single-molecule junctions (illustrated in Figure 5b). We conclude that while **Azo-NH**_**2**_ forms stable complexes with α-CD in our experiments, **Sti-NH**_**2**_ likely forms a less stable one,^78–80^ and we do not find any evidence for such **Sti-NH**_**2**_/α-CD complex bound to electrodes in forming single-molecule junctions.

To determine if the terminal group affects the formation as well as the molecular conductance of the host–guest complex, we synthesize **Azo-SMe**, an azobenzene derivative functionalized with thiomethyl anchoring groups (chemical structure is shown in Figure 6a), following previously published methods.^81^ Figure 6c displays the 1D conductance histograms of **Azo-SMe** measured in TCB, in H_2_O, and with addition of 40 equiv of α-CD in H_2_O. From the experiment performed in TCB, we find a single-molecule junction conductance of ∼1 × 10^–3^ *G*_0_ for **Azo-SMe**, the same as that of **Azo-NH**_**2**_. Notably, due to the insolubility of **Azo-SMe** in H_2_O, no conductance peak was observed for measurements performed in H_2_O (Figures 6c and S28). Upon addition of 40 equiv of α-CD, **Azo-SMe** becomes soluble in H_2_O, likely enclosed inside the host ring driven by its hydrophobicity, strongly indicating that stable host–guest complexes are formed. However, comparing data of α-CD/**Azo-SMe** host–guest complex with that of **Azo-SMe**, we find that conductance remains the same at 1 × 10^–3^ *G*_0_ (Figure 6c–e).

**Figure 6:**
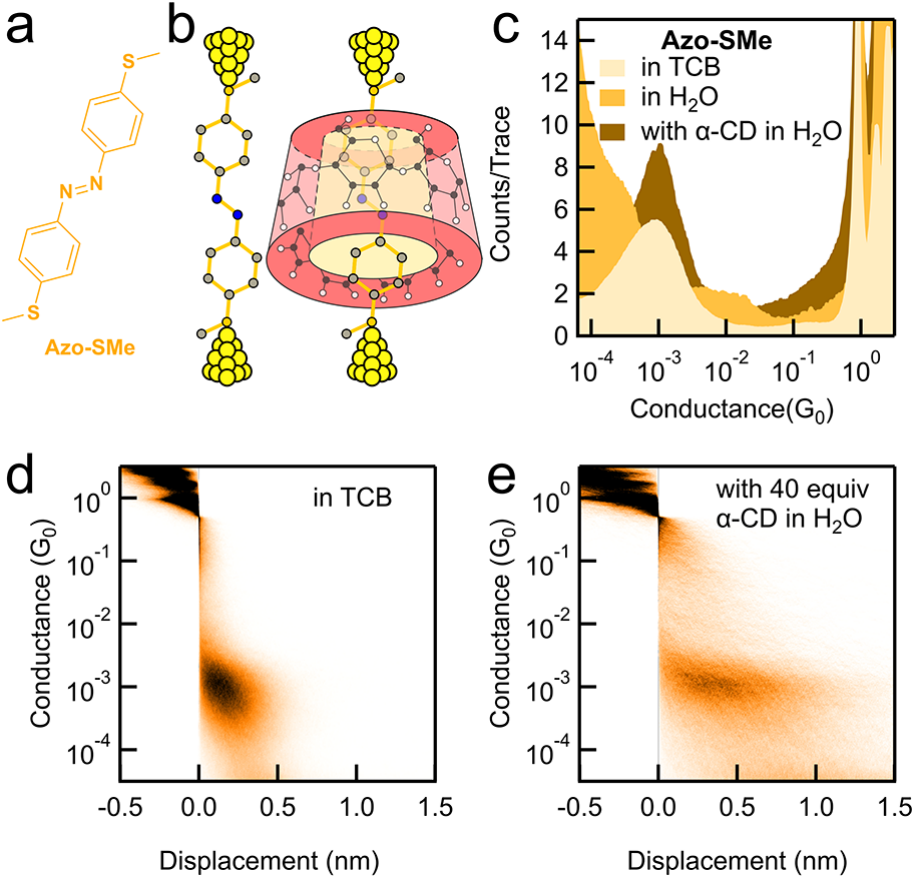
(a) Chemical structure of the guest molecule **Azo-SMe**. (b) Schematic representation of molecular junctions of **Azo-SMe** (left) and the **Azo-SMe**/α-CD complex (right). (c) 1D conductance histograms for **Azo-SMe** measured in TCB (0.1 mM), in H_2_O, and in H_2_O (0.5 mM) with the addition of 40 equiv of α-CD. **Azo-SMe** is not soluble in H_2_O; therefore, no single-molecule conductance peak is observed when H_2_O is used as the solvent. (d, e) 2D conductance histogram for **Azo-SMe** measured (d) in TCB and (e) in the presence of 40 equiv of α-CD in H_2_O.

In agreement with our experimental observations, our calculations indicate that the HOMO and LUMO energy levels for the α-CD/**Azo-SMe** host–guest complex are similar to those of **Azo-SMe** in the junction, and the overall transmission between the two frontier orbitals is similar for the complex and **Azo-SMe**. In addition, varied electronic properties of amine linkers have been observed when measurement conditions, such as the dielectric constant of the solvent and the applied voltage,^82,83^ or the presence of protons in the solvent,^70,71^ were regulated; thiomethyl linkers are resistive to such stimuli. This observation agrees with our general understanding of amines and thiomethyls.

## Conclusions

In this study, we investigate three guest molecules, amine-terminated azobenzene, amine-terminated stilbene, and thiomethyl-terminated azobenzene, and their corresponding α-CD-based host–guest systems, for understanding how host–guest interactions affect single-molecule junction charge transport. Single-molecule conductance measurements of amine-terminated azobenzene reveal a modest, approximately 3.5-fold enhancement upon host–guest complexation with cyclodextrin. DFT calculations suggest a shift of the LUMO toward the Fermi level upon formation of the α-CD/**Azo-NH**_**2**_ host–guest complex, leading to a slight increase in the electron transport for the complex junction in comparison to the guest junction. We find that although amine-terminated stilbene and thiomethyl-terminated azobenzene both share a similar structure with the amine-terminated azobenzene, the conductance of these two compounds remains the same upon the addition of α-CD, highlighting the critical roles played by both the linker and the backbone structure in regulating the host–guest complex junction conductance. Interestingly, the conductance of amine-terminated azobenzene/α-CD host–guest complexes shows a progressive decrease over time, which we hypothesize is primarily a result of the aggregation of the host molecules over time and ultimately is a measurement of only the uncomplexed guest molecules. In summary, this work establishes a fundamental understanding of the electronic properties of the azobenzene/cyclodextrin host–guest system, a classic supramolecular pair that is so far underexplored in molecular electronics. Our findings advance the design of cyclodextrin-based supramolecular chemical structures for use in electronic circuits and deepen our mechanistic understanding of the effects of noncovalent interactions on nanoscale charge transport.

## Supplementary Material



## Data Availability

Data for DFT calculations of frontier molecular orbitals and transport calculations using NEGF-DFT can be found from here: 10.5281/zenodo.18441724.

## Notes

Scanning tunneling microscope-based break-junction experiment details; Flicker noise experiment details; computational details; and additional Figures (S1–S28) and Tables (S1 and S2) (PDF)

## #

Friedrich Schiller University Jena, Lessingstrasse 4, 07743 Jena, Germany
