# Supplementary material for: Host–Guest Interactions Enhance Charge Transport across Single Cyclodextrin/Azobenzene Complex Junction
Source: J Am Chem Soc. 2026 Mar 12;148(11):11790–9. doi: 10.1021/jacs.5c20902 (PMC13022872; doi:10.1021/jacs.5c20902)
Supplement: Supplementary file 1 [file ja5c20902_si_001.pdf]

Supporting Information

Host-guest interactions enhance charge transport across single cyclodextrin/azobenzene complex junction

Song Han,† Jingjing Zhao,‡ Sumit Naskar,§,‖,# Di Wu,‡ Carmen Herrmann,§,‖ Jianlong Xia,‡,⊥ and Haixing Li †

†Department of Physics, City University of Hong Kong, Kowloon, Hong Kong 999077, China
‡State Key Laboratory of Advanced Technology for Materials Synthesis and Processing, Center of Smart Materials and Devices, and School of Chemistry, Chemical Engineering and Life Science, Wuhan University of Technology, Wuhan 430070, China
§Department of Chemistry, University of Hamburg, Harbor Bldg. 610, Luruper Chaussee 149, 22761 Hamburg, Germany
‖The Hamburg Centre for Ultrafast Imaging, University of Hamburg , Luruper Chaussee 149, 22761 Hamburg, Germany
⊥International School of Materials Science and Engineering, Wuhan University of Technology , Wuhan 430070, China
#Present Addresses:
S.N.: Friedrich Schiller University Jena, Lessingstrasse 4, 07743, Jena, Germany

Table of Contents

I. Scanning Tunneling Microscope-based Break Junction Experiment Details............ 2
II. Flicker Noise Experiment Details.. 3
III. Computational Details 5
IV. Additional Figures and Tables.. 7
V. References 30

I. Scanning Tunneling Microscope-based Break Junction Experiment Details

Scanning tunneling microscope-based break junction (STM-BJ) technique was used to carry out the single-molecule conductance experiments under ambient conditions. The detail of our experimental setup is provided in the supporting information of a previous work.[1] Specifically, Au tips were moved in and out of contact with a gold-coated steel substrate to form and break an Au–Au atomic contact. For the experiments performed in aqueous solution, the Au tips were coated with Apiezon wax to ensure that only the apex of the tip was exposed to make contact with the substrate. When the Au tip was withdrawn from the substrate, conductance as a function of the distance between the tip and the substrate was recorded.

In our measurements of solutions containing host and guest molecules in water, we observe a rapid conductance change accompanied by solvent evaporation. We note that aggregates of the host molecule are likely formed during the measurement,[2, 3] which potentially results in this conductance change. In order to reduce the impact of aggregates formation during the measurement, only around 1000 traces were collected as we observed a complete evaporation of one drop of host and guest mixture solution during this time period. Immediately after, we moved the wax-coated tip to a new contact area of the same substrate and added a drop of solution to continue the measurement. In the end, all recorded traces were compiled to construct conductance histograms shown in Figure 1d, 1f, and 2c in the manuscript (experiments of **Azo-NH$_2$** and α-CD molecular solution).

II. Flicker Noise Experiment Details

To gain deeper insights into the distinctions between the **Azo-NH$_2$** and **Azo-NH$_2$**/α-CD system, we conducted flicker noise analysis.[4] This method examines the relationship between the power spectrum density (PSD) and the average conductance (G_{ave}), expressed as PSD ∝ G_{ave}^n, and offers valuable information regarding the coupling at the molecule–electrode interface. Flicker noise is thought to originate primarily from fluctuations in the electronic coupling strength Γ between the molecule and the electrodes. Such fluctuations have been attributed in prior studies to atomic-scale rearrangements of the metal electrodes and changes in binding geometry and molecular conformation during one break-junction measurement.[4]

To determine the average conductance and power spectrum density, we apply an altered sequence of motion to the tip in each break-junction measurement. Specifically, after an initial withdrawal, the tip was held stationary for 100 ms at a displacement favoring the formation of a molecular junction, followed by a second withdrawal until the junction breaks and the two electrodes are separated. Thousands of single conductance traces under such modified sequence of motion to the tip were acquired under a 100 mV tip bias at a sampling rate of 100 kHz.

In our analysis, we first determine the average conductance of the junction G_{ave} during the entire 100 ms hold period and the average conductance of the junction during the last 50 ms of the hold period for each conductance trace. If both these average conductance values fall within the conductance peak range of the corresponding molecular junction, then the trace is selected for flicker noise analysis. The square of the integral of the discrete Fourier transform between 100 Hz and 1000 Hz of the conductance data collected during the 100 ms hold period is calculated as the PSD. In other words, the conductance noise power spectral density (PSD) for each junction is derived from the data recorded during the fixed displacement segment. Figures S1a–S1d show two example single traces along with their corresponding PSDs for a host–guest complex junction. Figure S1e displays the conductance PSD, averaged over thousands of such junctions, for the host–guest system under investigation.

For the selection of molecular junctions, the conductance range for **Azo-NH$_2$** in TCB is $3.5 \times 10^{-4} \sim 3.5 \times 10^{-3}$ G_0, for **Azo-NH$_2$**/α-CD lowG is $2.0 \times 10^{-4} \sim 2.0 \times 10^{-3}$ G_0, and for **Azo-NH$_2$**/α-CD highG is $2.0 \times 10^{-3} \sim 6.3 \times 10^{-2}$ G_0. After performing a discrete Fourier transform on the data and then squaring the result to obtain the PSD, we scale the PSD with G_{ave}^n on the data from all selected junctions. When a certain n leads to zero correlation between the PSD/G_{ave}^n and G_{ave}, the value n is the scaling exponent. We display 2D histograms of the normalized noise power (PSD/G_{ave}) against average conductance (G_{ave}).

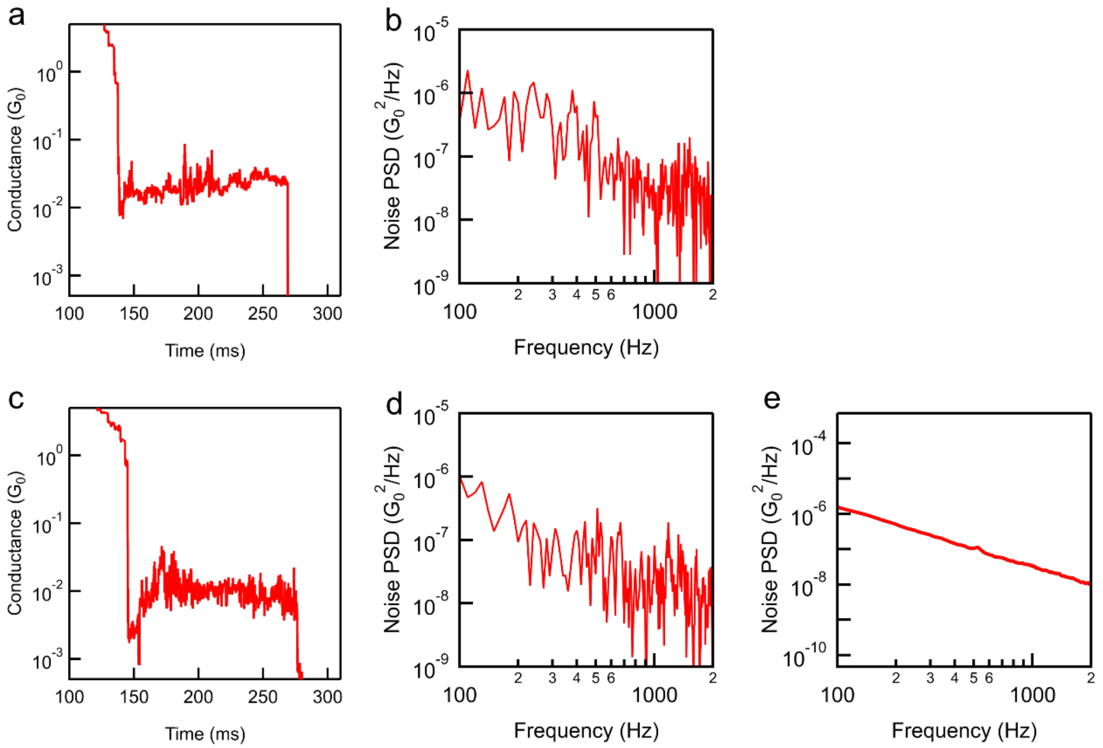

Figure S1. (a) Representative conductance versus time trace. (b) The conductance noise power spectral density obtained by taking the square of the discrete Fourier transform of the constant displacement section of the conductance trace in (a). (c) Representative conductance versus time trace. (d) The conductance noise power spectral density obtained by taking the square of the discrete Fourier transform of the constant displacement section of the conductance trace in (c). (e) Averaged conductance noise PSD measured for the **Azo-NH_2**/α-CD system.

III. Computational Details

Structures optimizations: Molecular structures of Azo-NH_2, α/Azo-NH_2, Azo-SMe, and α/Azo-SMe were optimized within Kohn-Sham density functional theory (DFT) where approximated exchange-correlation functional from BP86[5] with Ahlrichs' def2-TZVP basis set[6] were used. The atoms were relaxed until the change in the total energy of the self-consistent algorithm reaches 10^{-6} a.u. and the gradient of total energy with respect to nuclear displacement reaches 5×10^{-5} a.u. During optimization conductor like polarizable solvation model (CPCM)[7] was used with water as solvent and we used Grimme's D3 dispersion correction with Becke-Johnson damping[8]. After the optimization single-point energy calculations were carried out using hybrid B3LYP exchange correlation functional[9, 10] with keeping the other setting same as optimizations to obtained the MOs in Figure S17 and S23. All the calculations are performed with ORCA 5.01 program package.[11]

Junction structures: The optimized molecules are attached to two Au (111) gold clusters each of those contains 10 gold atoms with Au-Au bond lengths 2.88 Å same as the experimental bond lengths of bulk Au.[12] The Au-N distances were kept 2.35 Å as obtained from previously obtained values from DFT calculations which is close to the experimentally found bond length of 2.4 Å.[13, 14] The junction geometries can be seen in Figure S18 (-NH_2) and Figure S22 (-SMe). For -SMe anchoring groups we used Au-S distances 2.58 Å and 2.92 Å for two different junction geometries.

Transmission functions calculations: To evaluate transmission functions single-point energy calculations were carried out for the junction structures with the same SCF convergence threshold used for structure optimizations. The calculations for the junctions were done using hybrid B3LYP exchange correlation functional and def2-TZVP basis set. All these calculations were carried out in ORCA 5.01 program package.[11]

The effective single-particle Hamiltonian and overlap matrices are extracted using ARTAIOS postprocessing program package.[15, 16] For evaluation of transmission function we used Landauer-Bütiiker-Imry formalism combined with Green's function. The transmission function in that formalism is written as:

$$T_{RL} = Tr[\Gamma_L G_L \Gamma_R G_R^\dagger] \quad (1)$$

$$G = (ES_C - H_C - \Sigma_L - \Sigma_R)^{-1} \quad (2)$$

where Eqn:2 represents Green's function of the central region which contains only the molecules. E is the energy, S_C and H_C are the overlap and Hamiltonian matrices of the central region expressed in the atom-centered basis (overlap and Fock matrices). The coupling of the central region molecules to the gold clusters (electrodes) are achieved using a bulk broadening function written as:

$$\Gamma_X = -2\mathrm{Im}\ \Sigma_X \quad (3)$$

where Σ_X are the self energies of left or right electrode (X=L/R). Σ_X can be evaluated using the Hamiltonian and overlap matrices between the central region and the electrode region:

$$\Sigma_X = (ES_{XC}-H_{XC})^{\dagger}\ g_X\ \ (ES_{XC}-H_{XC}) \quad (4)$$

where g_X is the electrode's (L/R) Green's function, the elements of g_X can be evaluated using a constant local density of sates ($LDOS^{const.}$) as:

$$(g_X)_{ij} = -i\pi LDOS^{const.}\delta_{ij} \quad (5)$$

where according to wide-band limit $LDOS^{const.} = 0.036\ eV^{-1}$ as obtained from DFT results for 6s band of gold,[17] assuming the self energy as purely imaginary quantity.

The MO energies are evaluated by solving secular equation of the central subsystem only by using the equation:

$$H_C C_C = S_C C_C \varepsilon_C \quad (6)$$

where C_C and ε_C are the central subsystem MO coefficients and the subsystem matrix of Lagrange's multipliers (MO energies), respectively. Identification of MOs were done by comparing the shape of the subsystem MOs to the isolated molecules MOs where they are close in energies. The order of MOs can change going from isolated molecules to the junctions. In those cases the MOs are assigned in such a way where HOMO-LUMO gap is minimum. The subsystem MOs can be seen in Figure S20.

IV. Additional Figures and Tables

Table S1. Details for the 1D histograms presented in this work.

Dataset‡	Entry	Number of traces	Tip withdrawing speed (nm/s)	Figure
1	1*	15200	18	1d (in H_2O), 2c (0 eq.)
2	1	12000	18	1d (in TCB)
3	1*	8800	30	1d (in H_2O with α-CD), 2c (40 eq.)
4	1	400	18	part of 2a (25 m), S6 (25 m)
	2	400	18	part of 2a (50 m), S6 (50 m)
	3	400	18	part of 2a (75 m), S6 (75 m)
	4	300	18	part of 2a (100 m), S6 (100 m)
5	1	500	18	part of 2a (25 m), S7 (25 m)
	2	500	18	part of 2a (50 m), S7 (50 m)
	3	500	18	part of 2a (75 m), S7 (75 m)
	4	600	18	part of 2a (100 m), S7 (100 m)
6	1	1000	18	part of 2a (25 m), S8 (25 m)
	2	1000	18	part of 2a (50 m), S8 (50 m)
	3	1000	18	part of 2a (75 m), S8 (75 m)
	4	800	18	part of 2a (100 m), S8 (100 m)
7	1	800	18	part of 2a (25 m), S9 (25 m)
	2	800	18	part of 2a (50 m), S9 (50 m)
	3	800	18	part of 2a (75 m), S9 (75 m)
	4	700	18	part of 2a (100 m), S9 (100 m)
8	1*	7400	30	2c (20 eq.)
9	1	6200	12	5c (in TCB)
10	1*	7300	18	5c (in H_2O)
11	1*	10000	30	5c (in H_2O with α-CD)
12	1	20000	18	6c (in TCB)
13	1*	3500	30	6c (in H_2O)
14	1*	7600	22.5	6c (in H_2O with α-CD)
15	1	5800	19.2	S3
16	1	1000	18	S4
17	1	200	32	S12 (0-20 min)
	2	300	32	S12 (20-40 min)
	3	9500	32	S12 (40-650 min)
18	1*	1800	18	S13a
19	1*	600	30	S13c

20	1*	4400	32	S14a
21	1*	2300	32	S15a

‡All experiments were performed under a tip bias voltage of 100 mV.

*In order to reduce the impact of aggregates formation during the measurement, after every ~1000 traces were collected, immediately after, we moved the wax-coated tip to a new contact area of the same substrate and added a drop of solution to continue the measurement of the next 1000 junctions. In the end, all recorded traces were compiled to construct conductance histograms.

Table S2. Details for the 2D histograms presented in this work.

Dataset ‡	Entry	Number of traces	Tip withdrawing speed (nm/s)	Figure
1	1	12000	18	1e
2	1*	8800	30	1f
3	1	3100	18	2b
4	1	400	18	part of S10a, part of 2d (25m), S6 (25 m)
	2	400	18	part of S10a, part of 2d (50m), S6 (50 m)
	3	400	18	part of S10a, part of 2d (75m), S6 (75 m)
	4	300	18	part of S10a, part of 2d (100m), S6 (100 m)
5	1	500	18	part of S10b, part of 2d (25m), S7 (25 m)
	2	500	18	part of S10b, part of 2d (50m), S7 (50 m)
	3	500	18	part of S10b, part of 2d (75m), S7 (75 m)
	4	600	18	part of S10b, part of 2d (100m), S7 (100 m)
6	1	1000	18	part of S10c, part of 2d (25m), S8 (25 m)
	2	1000	18	part of S10c, part of 2d (50m), S8 (50 m)
	3	1000	18	part of S10c, part of 2d (75m), S8 (75 m)
	4	800	18	part of S10c, part of 2d (100m), S8 (100 m)
7	1	800	18	part of 2b, part of 2d (25m), S9 (25 m)
	2	800	18	part of 2b, part of 2d (50m), S9 (50 m)
	3	800	18	part of 2b, part of 2d (75m), S9 (75 m)
	4	700	18	part of 2b, part of 2d (100m), S9 (100 m)
8	1	6200	12	5d
9	1*	10000	30	5e
10	1	20000	18	6d
11	1*	7600	22.5	6e
12	1	15200	18	S2
13	1	5800	19.2	S3
14	1	1000	18	S4
15	1	1500	18	S10a
16	1	2100	18	S10b
17	1	3800	18	S10c
18	1*	1800	18	S13b
19	1*	600	30	S13d
20	1*	4400	32	S14b

21	1*	2300	32	S15b
22	1	7300	18	S26
23	1	3500	30	S28

‡All experiments were performed under a tip bias voltage of 100 mV.

*In order to reduce the impact of aggregates formation during the measurement, after every ~1000 traces were collected, immediately after, we moved the wax-coated tip to a new contact area of the same substrate and added a drop of solution to continue the measurement of the next 1000 junctions. In the end, all recorded traces were compiled to construct conductance histogram.

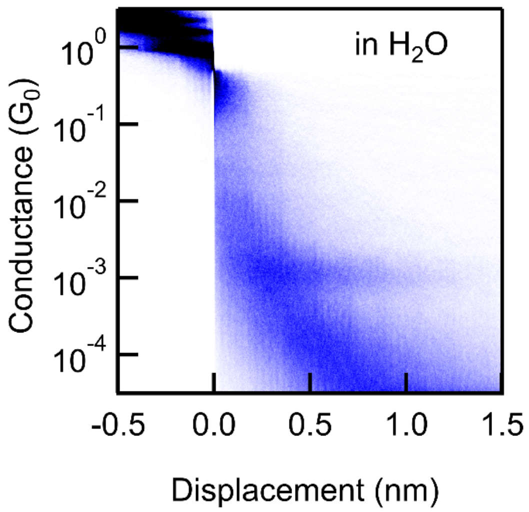

Figure S2. 2D conductance histogram of **Azo-NH2** measured in H_2O under 100 mV tip bias voltage.

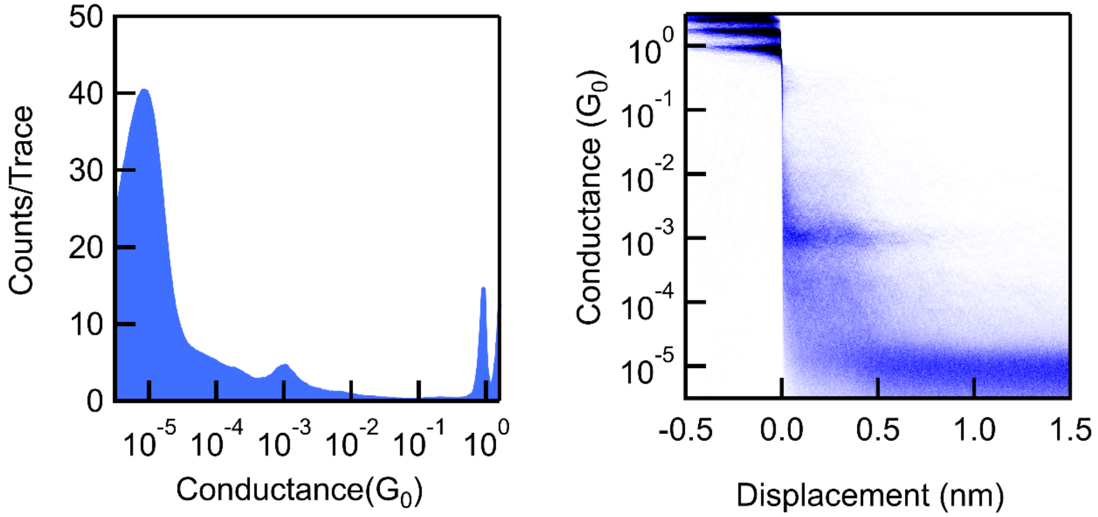

Figure S3. 1D and 2D conductance histograms of **Azo-NH2** in H_2O upon evaporation of the solvent (under a dry condition) with a non-coated tip under a 100 mV tip bias voltage. The instrument background noise is displayed in these histograms, and for a clear display of the data, this background noise is not shown in other histograms.

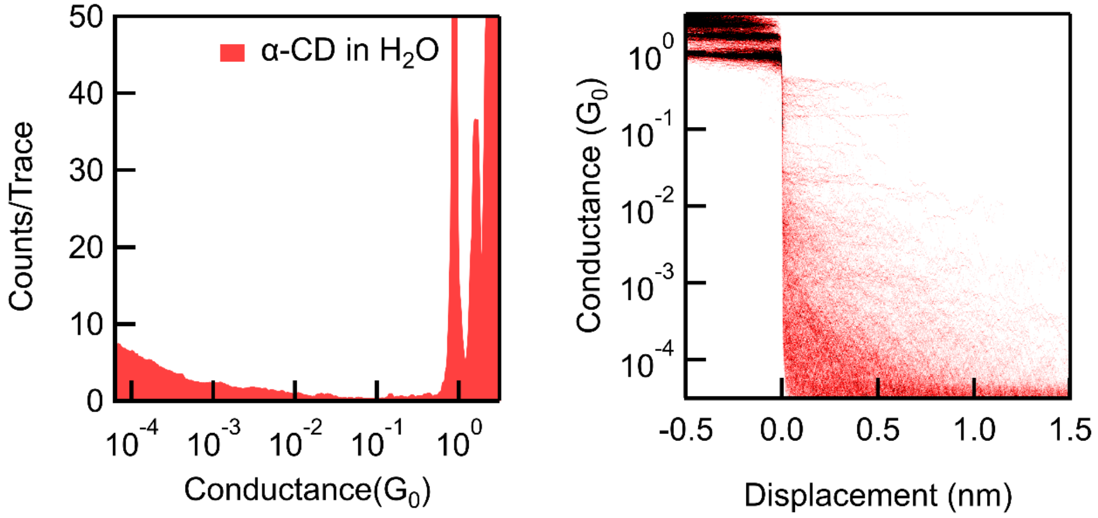

Figure S4. 1D and 2D conductance histograms of 40 mM α-CD measured in H_2O under a 100 mV tip bias voltage.

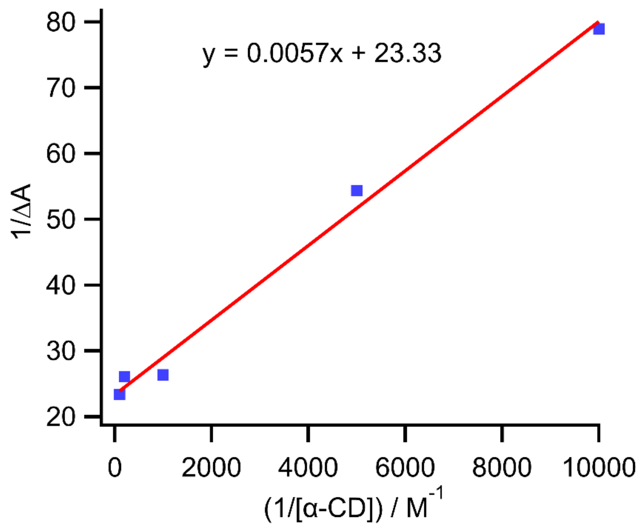

Figure S5. Double reciprocal plot of 1/Δ*A* as a function of 1/[α-CD], obtained based on the data shown in Figure 1g in the manuscript.

Discussion about Figure S5: The association constant between **Azo-NH$_2$** and α-CD in aqueous solution was determined by analyzing the UV/vis absorption peak at 398 nm, as shown in Figure 1g. The concentration of **Azo-NH$_2$** was kept at 1×10^{-5} M. Upon addition of excess α-CD, we see an increase in the absorption peak intensity at 398 nm. For a 1:1 complexation model, the host-guest complexation between α-CD(H) and **Azo-NH$_2$**(G) can be represented by the following equilibrium equation:

$$H + G \rightleftharpoons H \cdot G$$

We employed the double reciprocal plot according to the modified Hidebrand-Benesi equation:

$$\frac{1}{\Delta A} = \frac{1}{K_a \Delta\varepsilon [H][G]} + \frac{1}{\Delta\varepsilon [G]}$$

where [H,] [G], and K_a represent concentration of α-CD, concentration of **Azo-NH$_2$**, and association constant, respectively. ΔA denotes the absorbance difference between before and after α-CD was added. Δ*ε* represents the difference of the molar extinction coefficient between the host and host-guest complex at the same wavelength. The association constant K_a was calculated by the equation:

$$K_a = \frac{1}{k\Delta\varepsilon[G]} = 4.1 \times 10^3 M^{-1}$$

where *k* is the slope of the linear fit to the data in Figure S5.

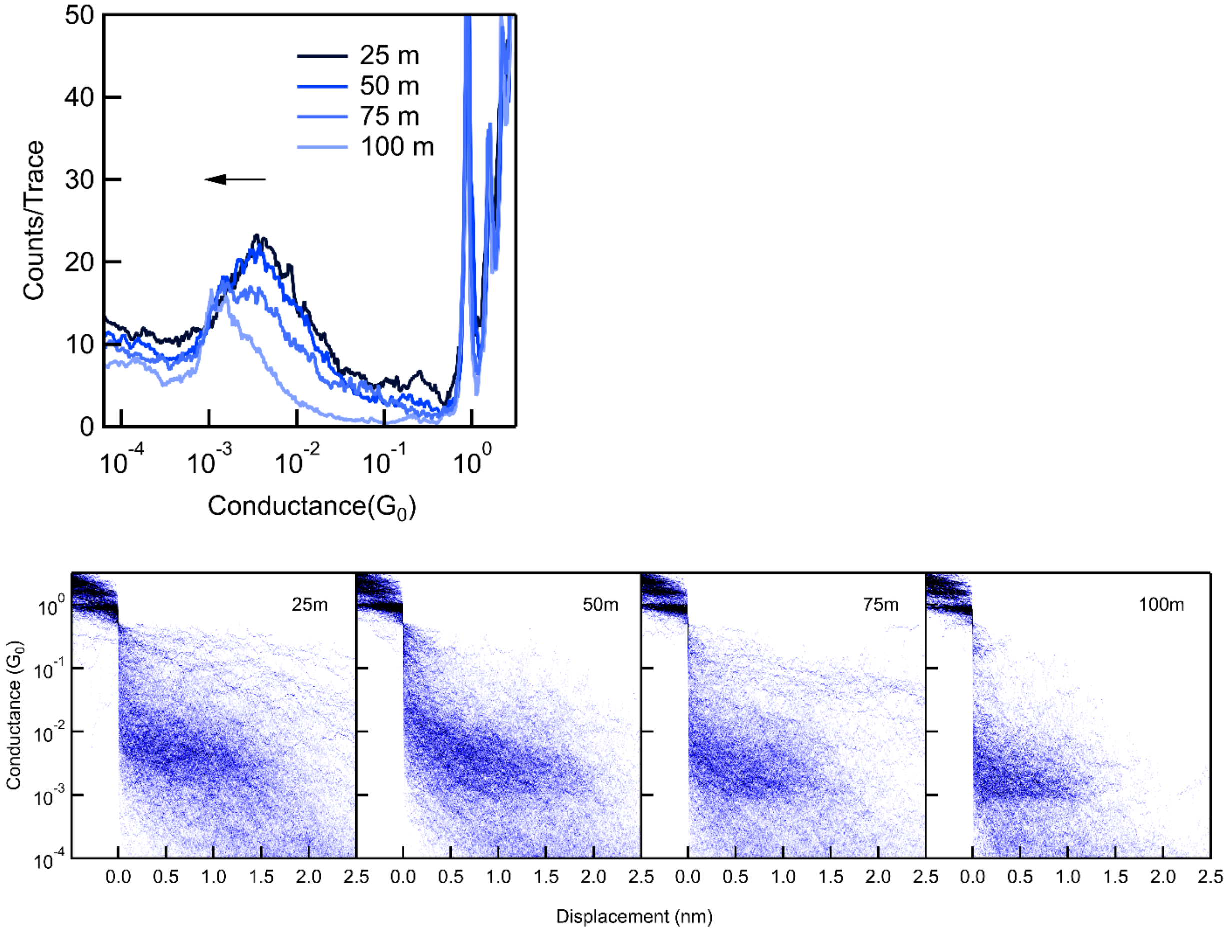

Figure S6. Data #1 of 1D and 2D conductance histograms of **Azo-NH₂** (1 mM) measured in the presence of 40 equivalents of α-CD in H_2O under a bias of 0.1 V in 0-25 mins, 25-50 mins, 50-75 mins, and 75-100 mins.

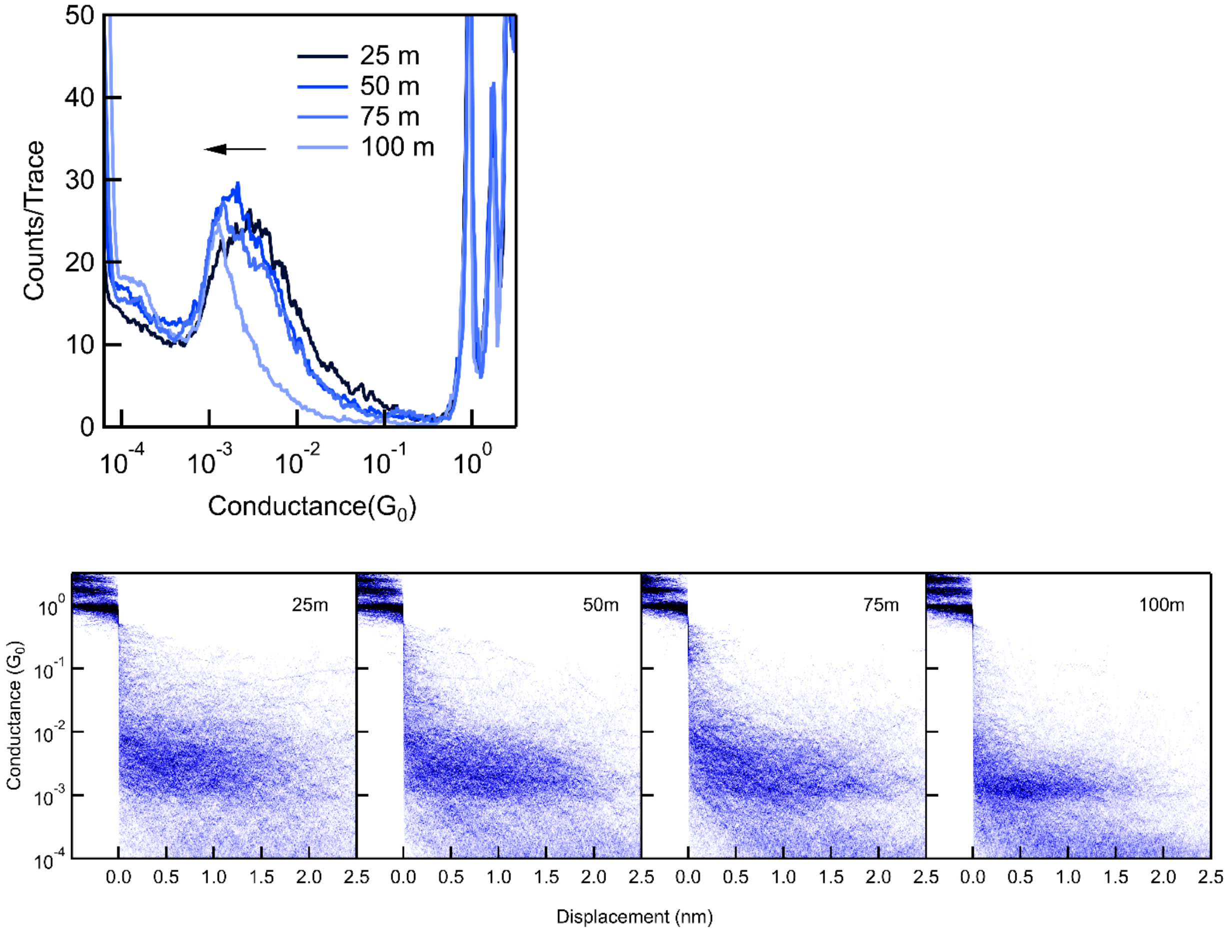

Figure S7. Data #2 of 1D and 2D conductance histograms of **Azo-NH$_2$** (1 mM) measured in the presence of 40 equivalents of α-CD in H_2O under a bias of 0.1 V in 0-25 mins, 25-50 mins, 50-75 mins, and 75-100 mins.

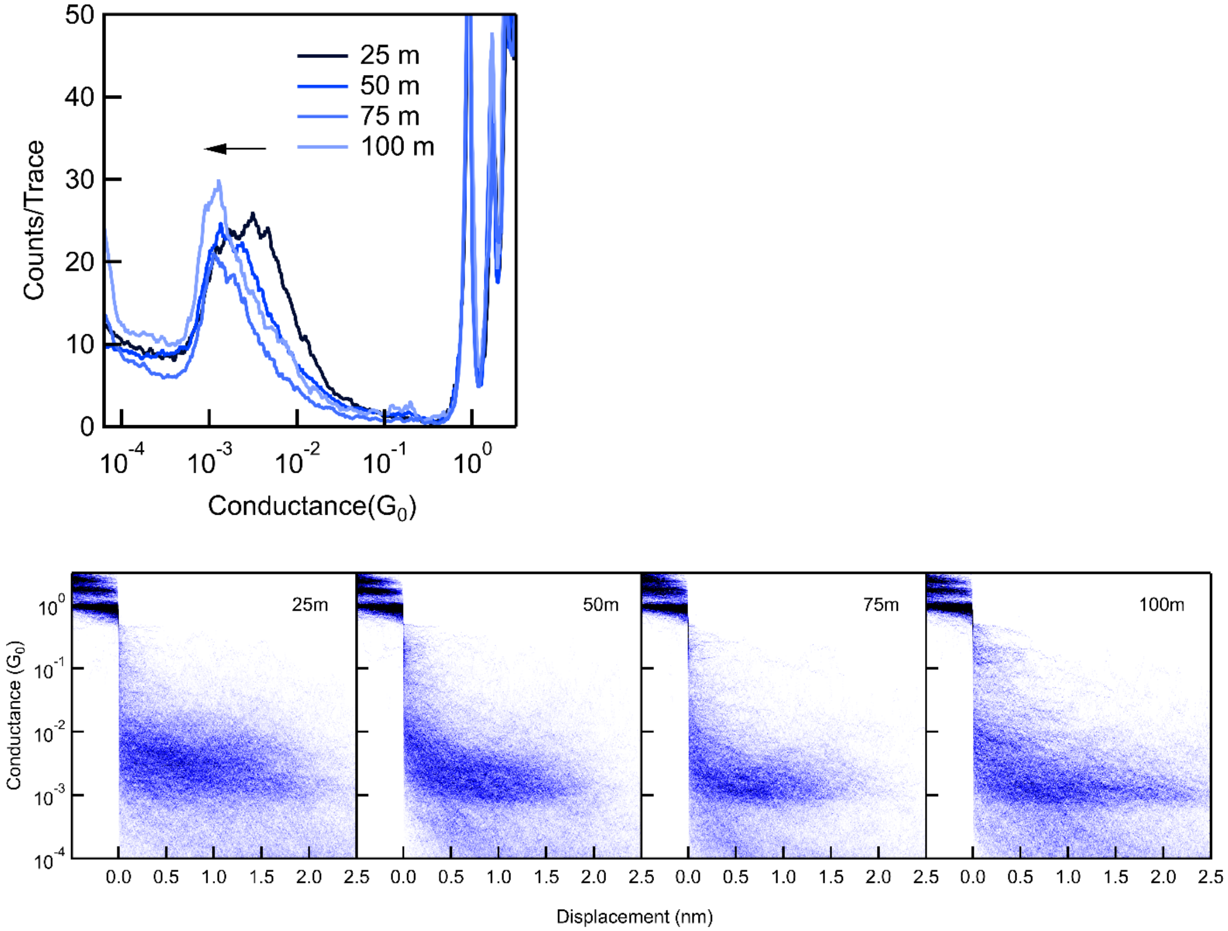

Figure S8. Data #3 of 1D and 2D conductance histograms of **Azo-NH2** (1 mM) measured in the presence of 40 equivalents of α-CD in H_2O under a bias of 0.1 V in 0-25 mins, 25-50 mins, 50-75 mins, and 75-100 mins.

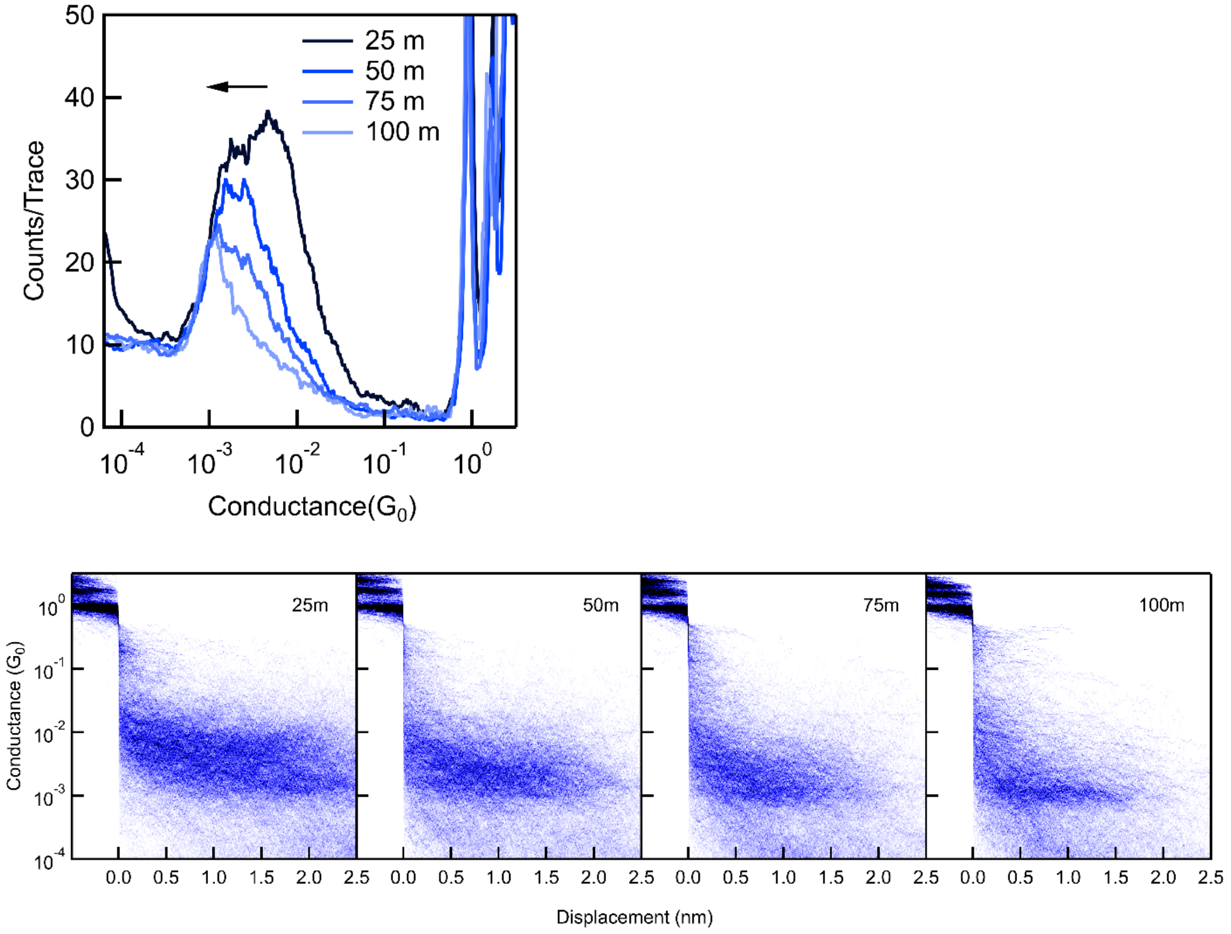

Figure S9. Data #4 of 1D and 2D conductance histograms of **Azo-NH$_2$** (1 mM) measured in the presence of 40 equivalents of α-CD in H_2O under a bias of 0.1 V in 0-25 mins, 25-50 mins, 50-75 mins, and 75-100 mins.

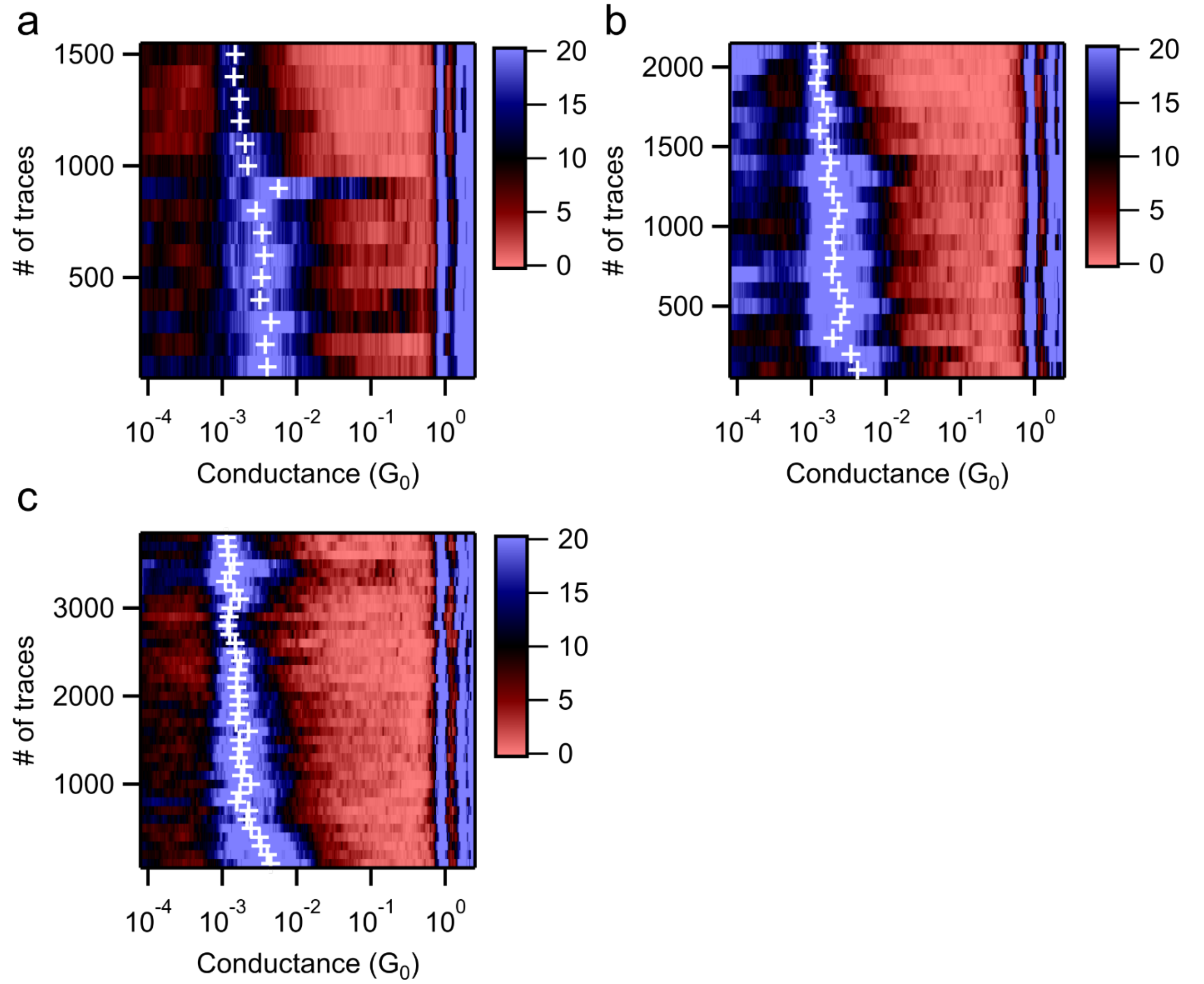

Figure S10. (a-c) Conductance as a function of time from 3 repeated experiments. The data collected from one other experiment is shown in Figure 2b in the manuscript.

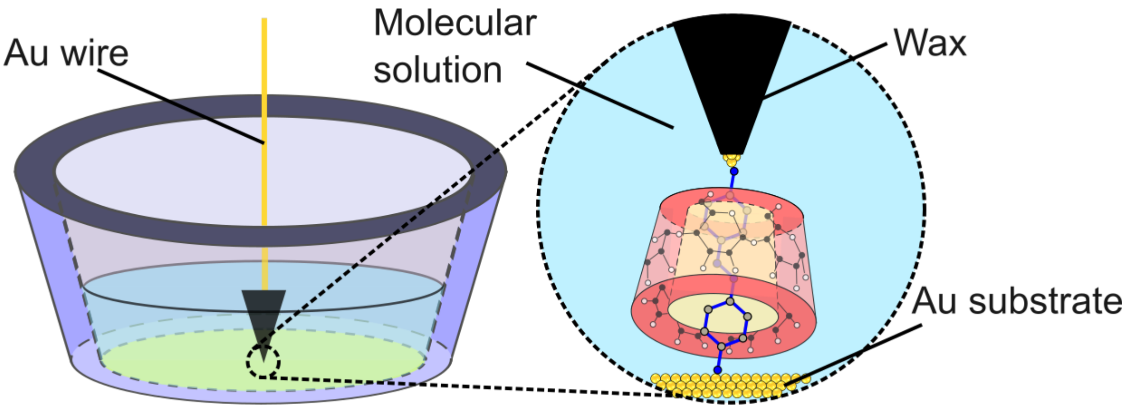

Figure S11. Schematic diagram of the custom-designed liquid cell for STM-BJ experiments.

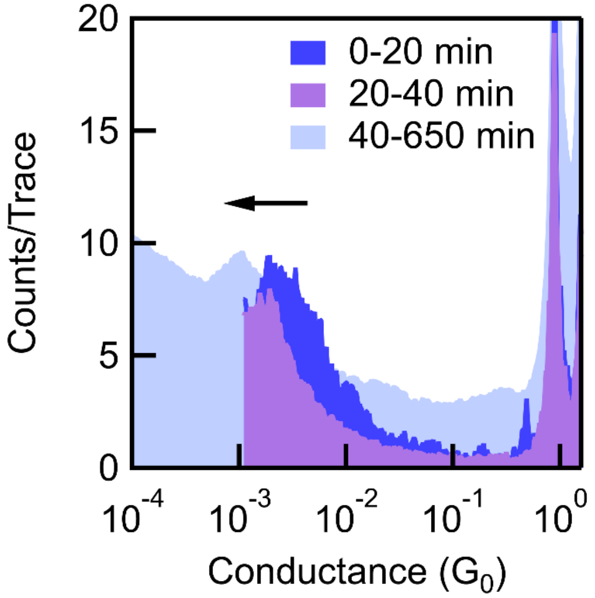

Figure S12. 1D conductance histograms of 0.2 mM **Azo-NH₂** measured in H_2O with addition of 40 equivalents of α-CD with a custom-designed liquid cell.

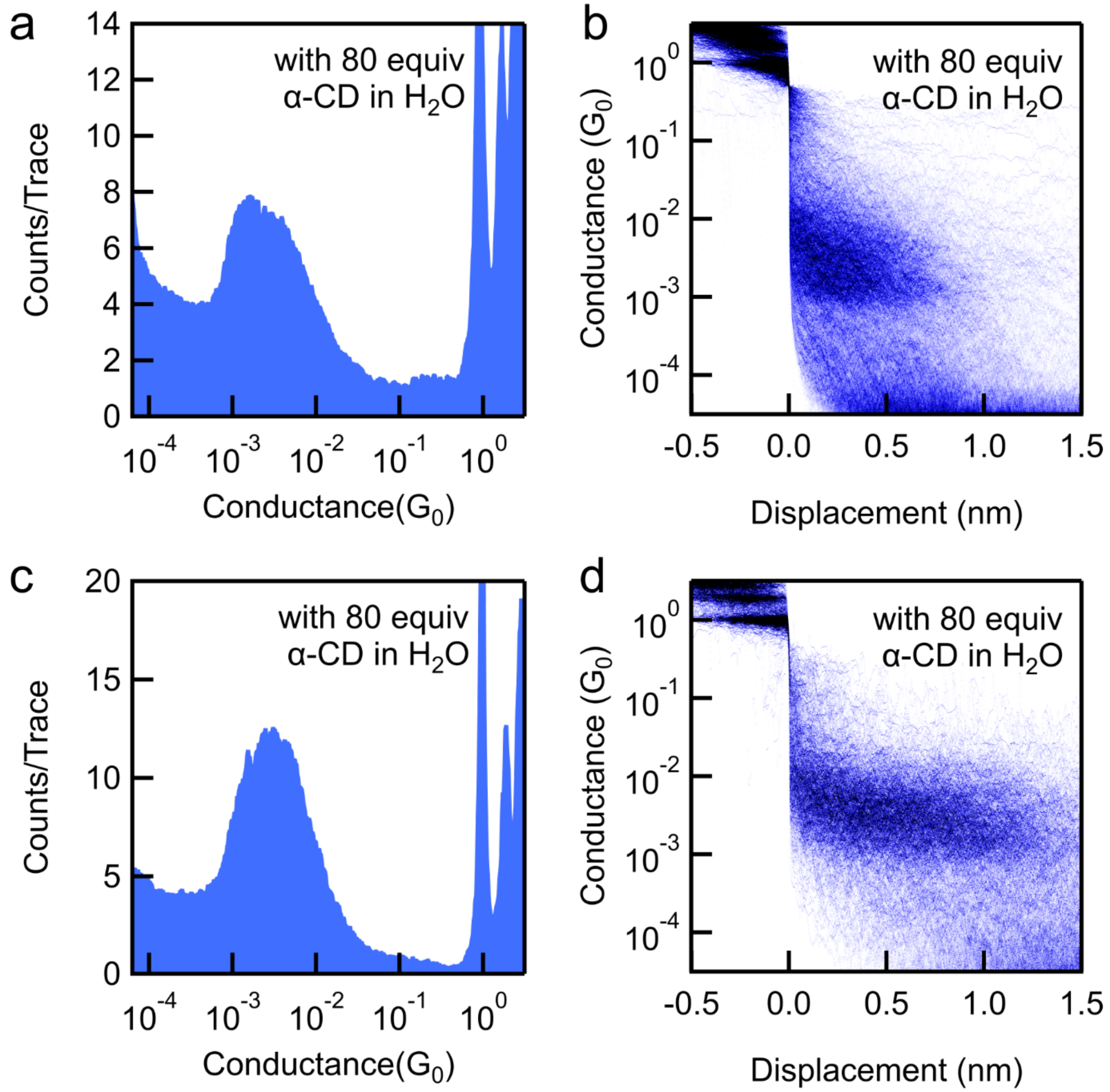

Figure S13. (a,c) 1D and (b,d) 2D conductance histograms of **Azo-NH₂**/α-CD from 2 repeated experiments. In each experiment, 0.2 mM **Azo-NH₂** in H_2O with addition of 80 equivalents of α-CD was measured under a 100 mV tip bias voltage. The tip withdrawing speed was 18nm/s for (a,b) and 30 nm/s for (c,d).

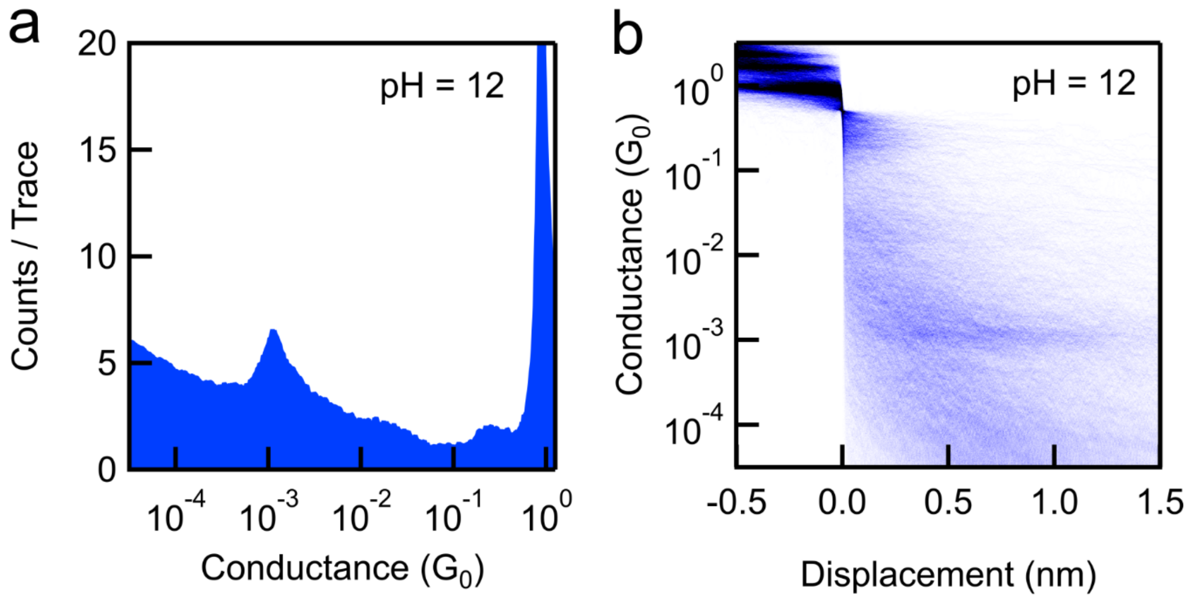

Figure S14. 1D & 2D conductance histograms of **Azo-NH_2** (0.2 mM) measured in sodium hydroxide aqueous solution with addition of 40 equivalents of α-CD at pH = 12.

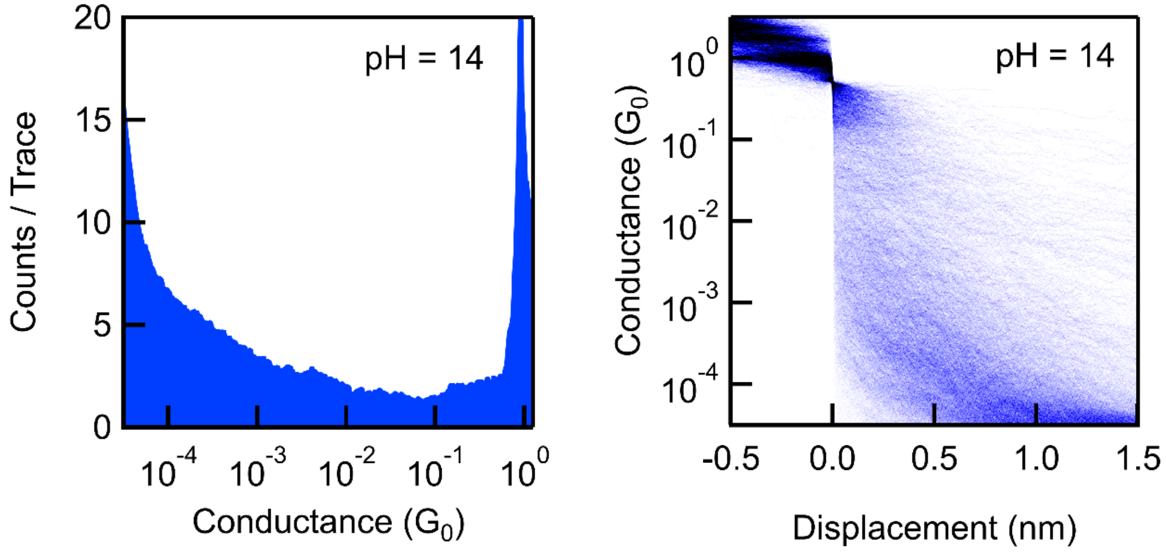

Figure S15. 1D & 2D conductance histograms of **Azo-NH_2** (0.2 mM) measured in sodium hydroxide aqueous solution with addition of 40 equivalents of α-CD at pH = 14.

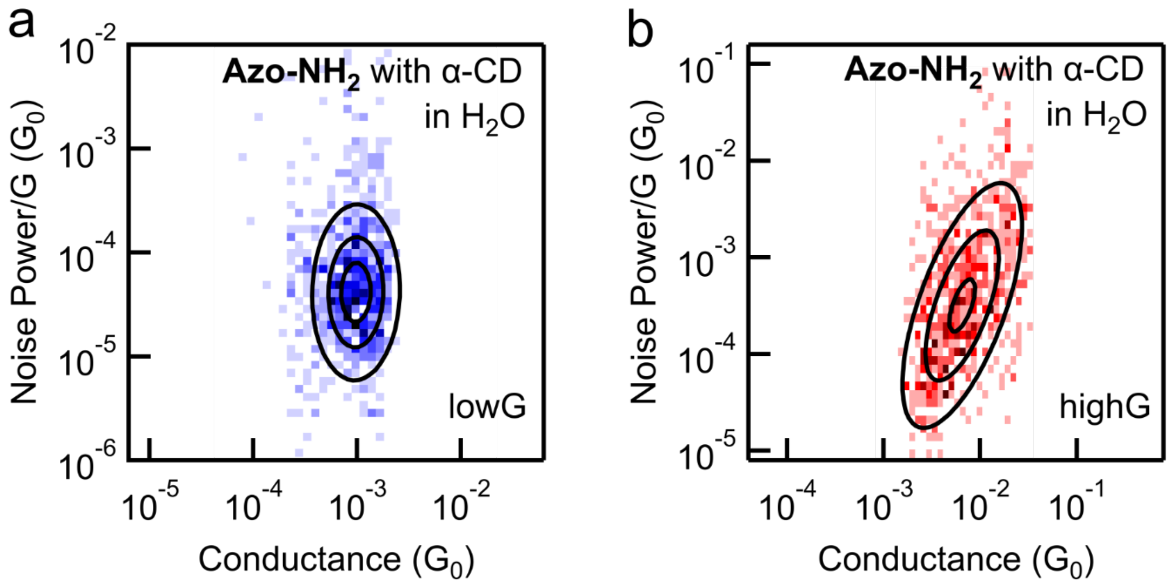

Figure S16. Repeated experiment of the one shown in Figure 3b and 3c in the manuscript. The scaling exponents determined for data in (a) and (b) are 1.07 and 2.31, in agreement with the results in Figure 3b and 3c in the manuscript.

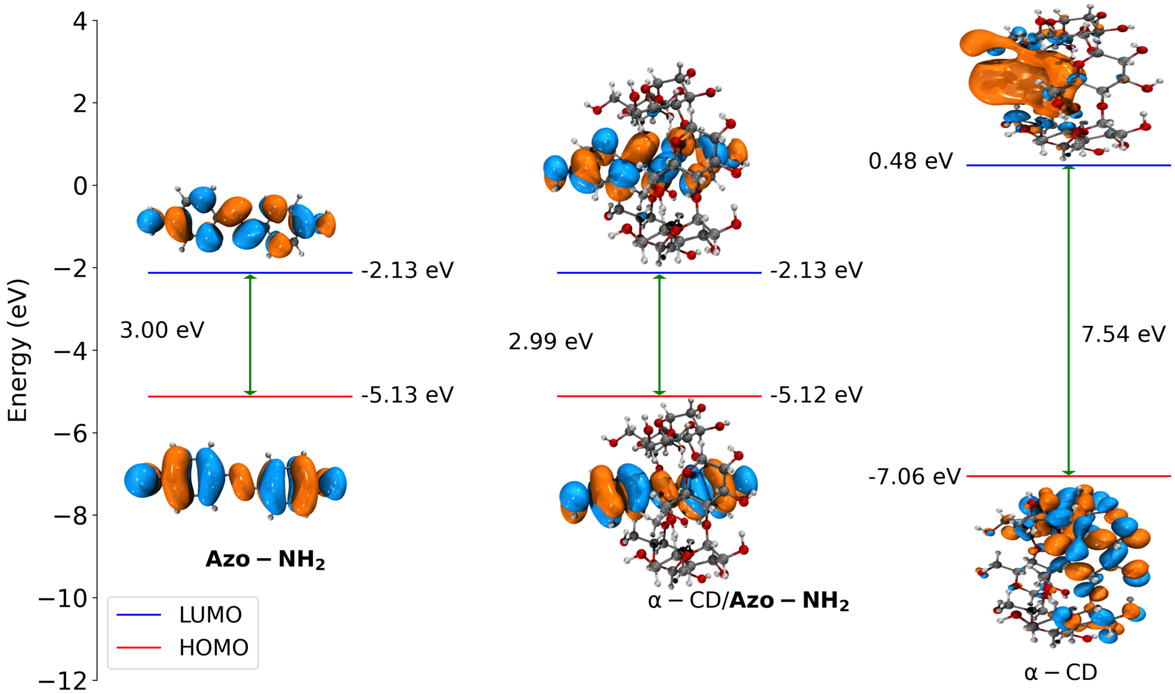

Figure S17. Energy levels diagram for frontier molecular orbitals of isolated **Azo-NH₂**, α-CD/**Azo-NH₂** complex, and α-CD calculated with B3LYP exchange correlation functional and def2-TZVP basis set.

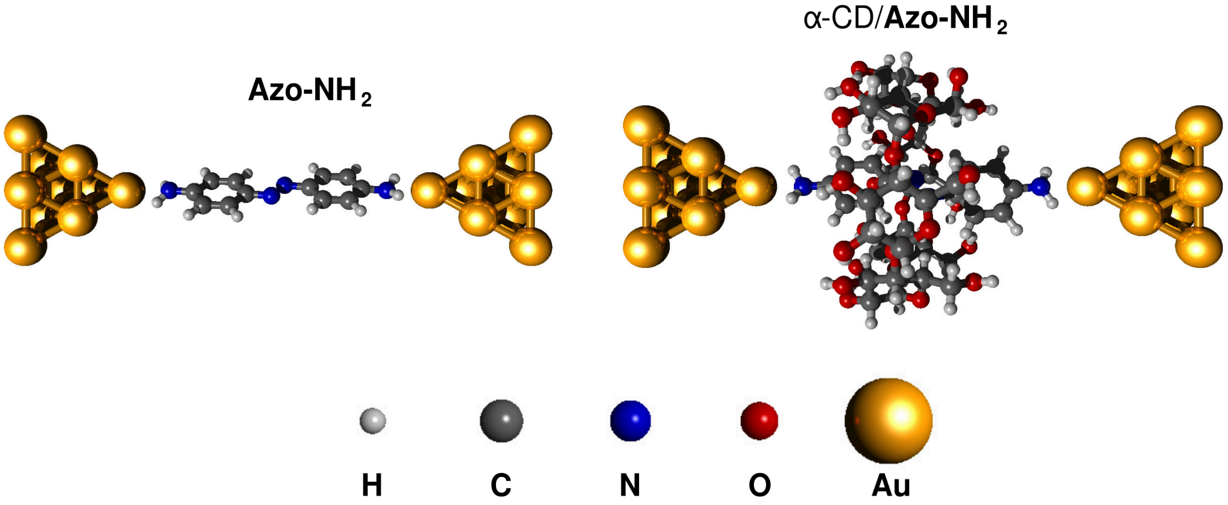

Figure S18. Device geometries of the **Azo-NH₂** and α-CD/**Azo-NH₂** complex junctions. Au-N distance is set to 2.35Å as obtained from previous reports.[13, 14]

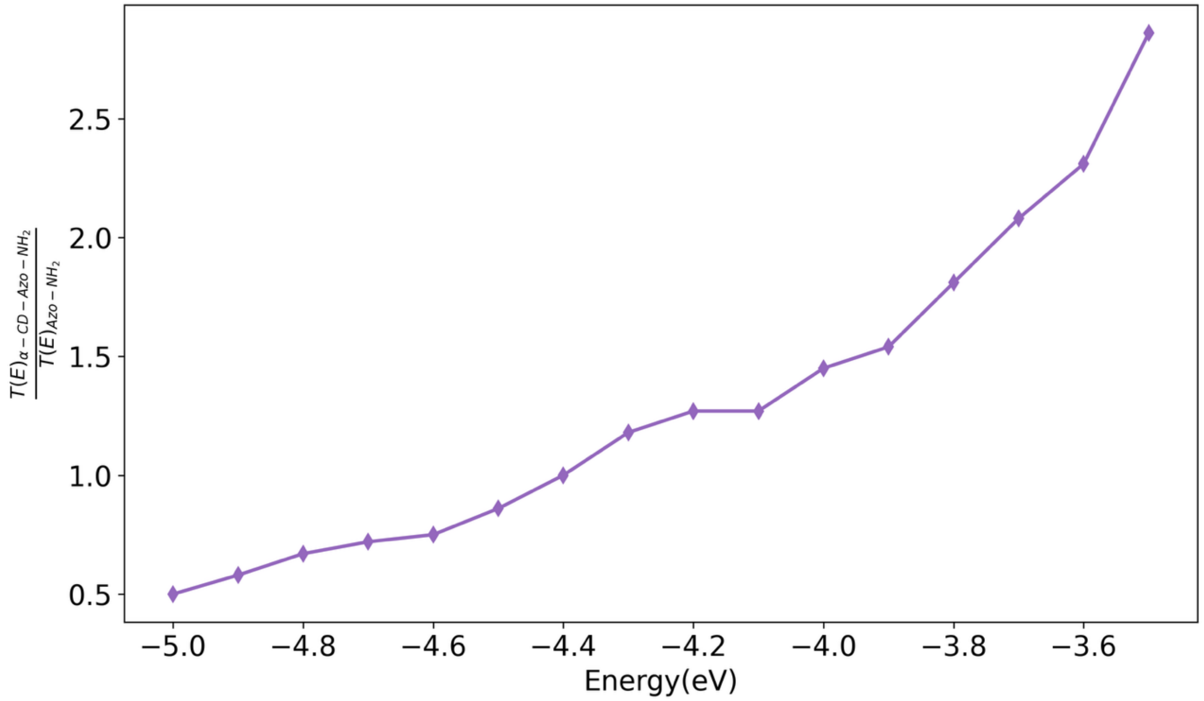

Figure S19. Estimated conductance change for α-CD/**Azo-NH₂** complex junctions in comparison to the **Azo-NH₂** junctions determined from transmission calculations shown in Figure 4 in the manuscript.

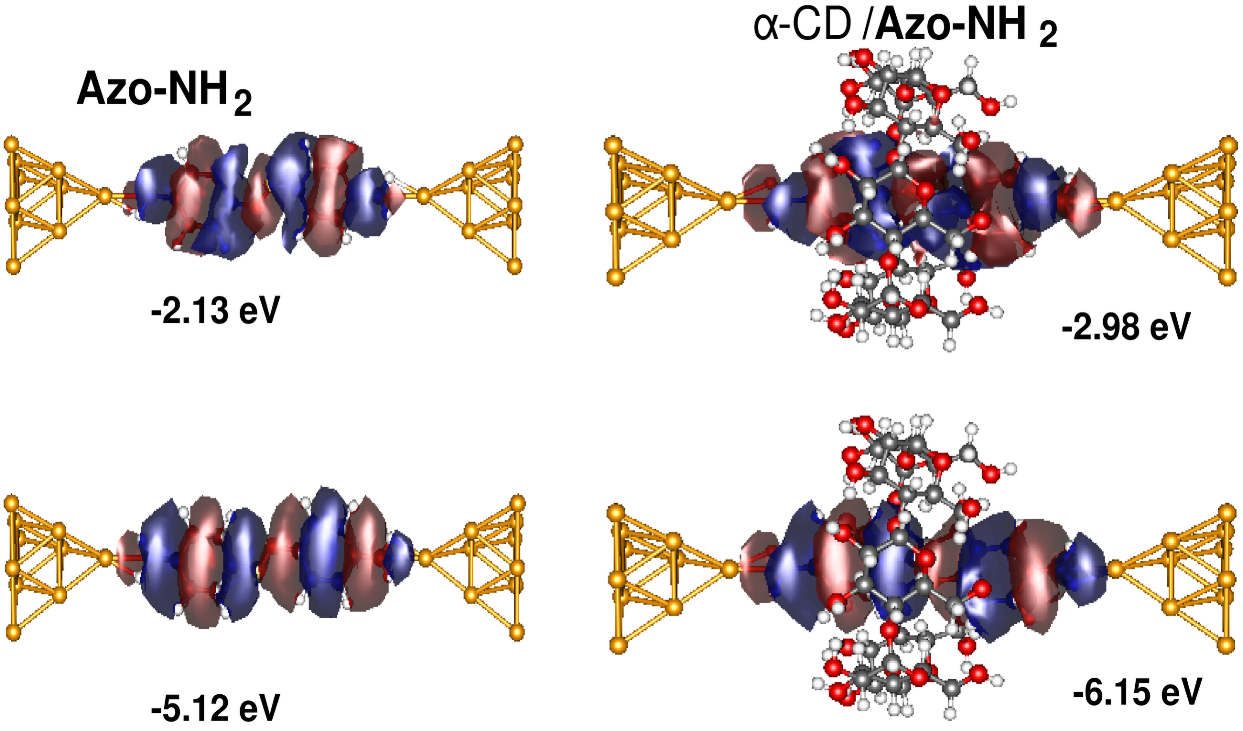

Figure S20. Central subsystem MOs for **Azo-NH₂** and α-CD/**Azo-NH₂** complex junctions calculated with B3LYP exchange correlation functional and def2-TZVP basis set.

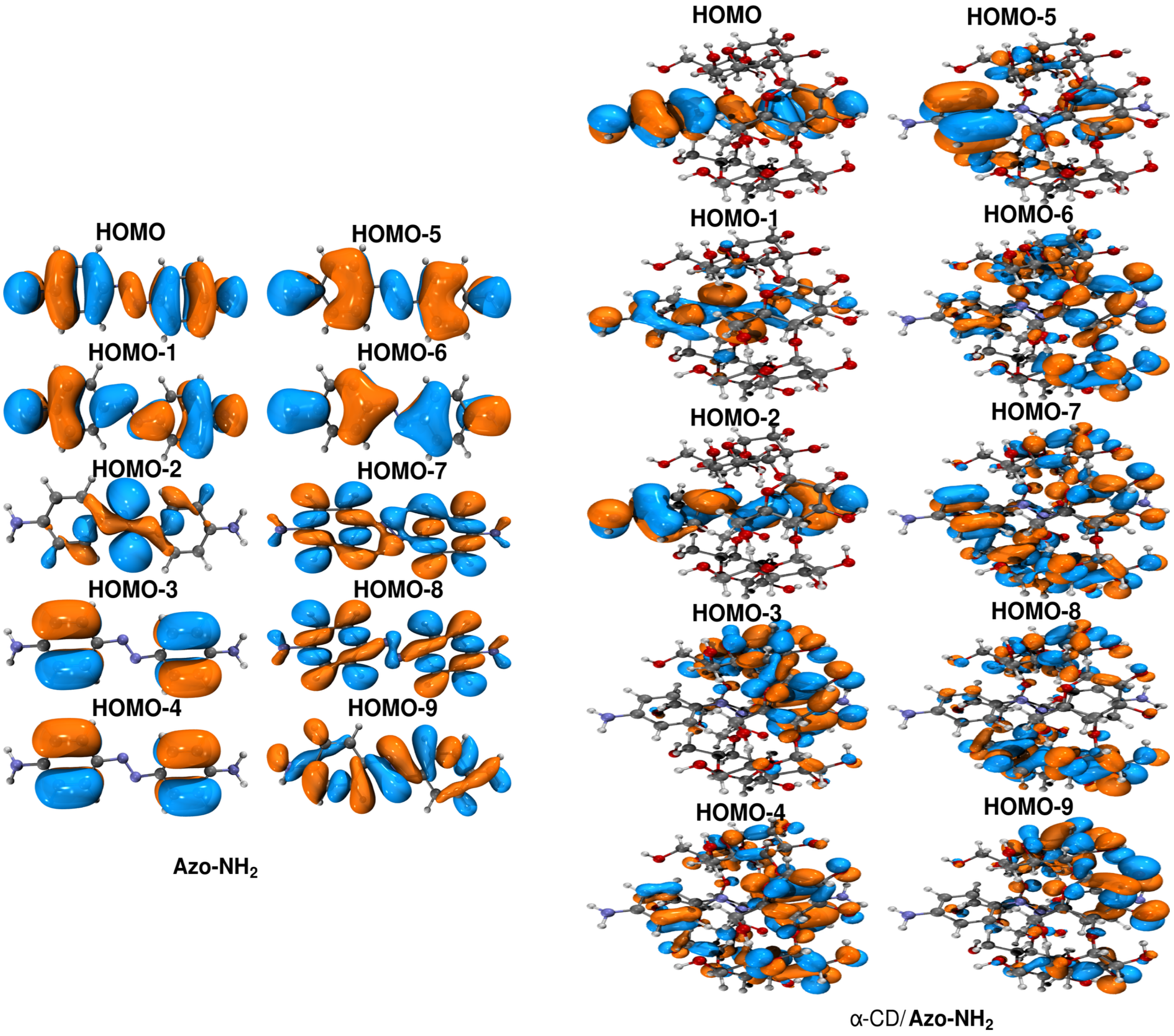

Figure S21. Lower energies occupied orbitals for the studied **Azo-NH_2** and α-CD/**Azo-NH_2** complex using B3LYP functional and def2-TZVP basis set.

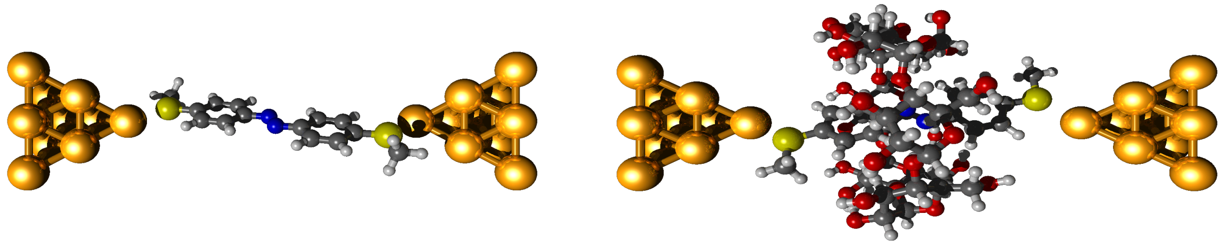

Figure S22. Device geometries of the **Azo-SMe** and α-CD/**Azo-SMe** complex junctions.

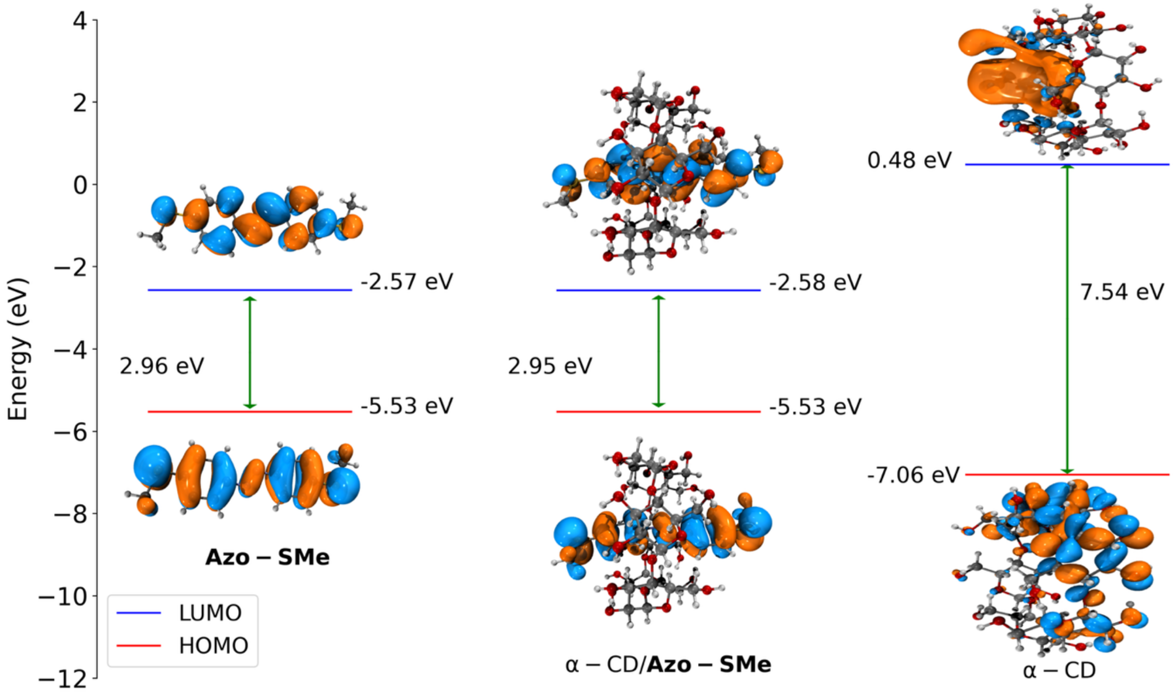

Figure S23. Energy levels diagram for frontier molecular orbitals of isolated **Azo-SMe**, α-CD/**Azo-SMe** complex, and α-CD calculated with B3LYP exchange correlation functional and def2-TZVP basis set.

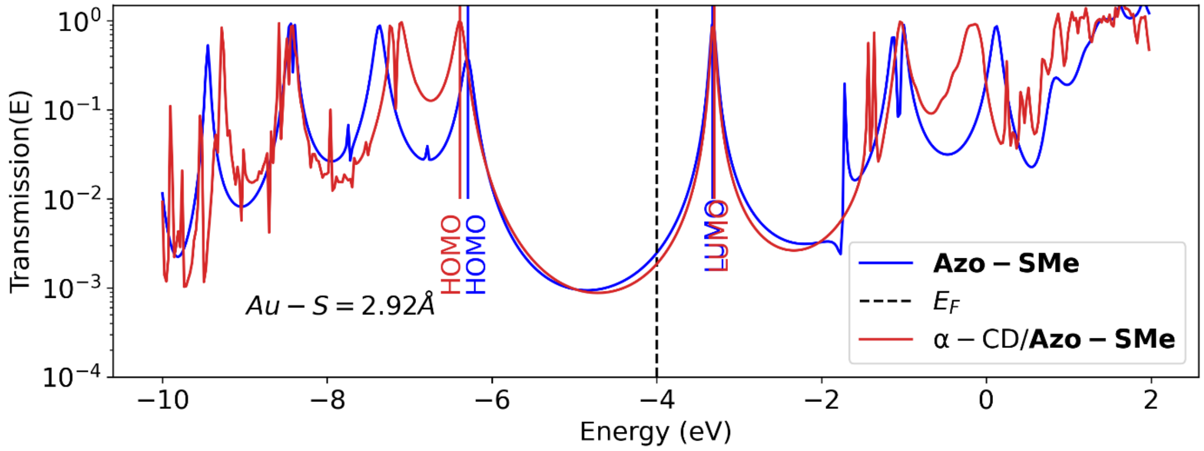

Figure S24. Transmission functions for **Azo-SMe** and α-CD/**Azo-SMe** junctions with Au-S distance of 2.92 Å. The transmission functions for the same junctions with Au-S distance of 2.58 Å are shown in the manuscript Figure 4.

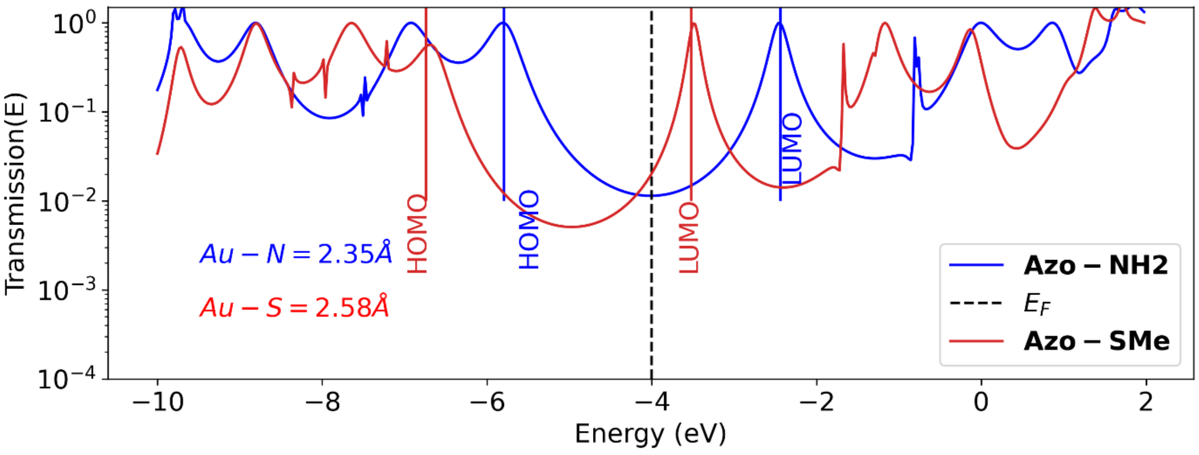

Figure S25. Transmission functions for **Azo-SMe** and **Azo-NH$_2$** junctions.

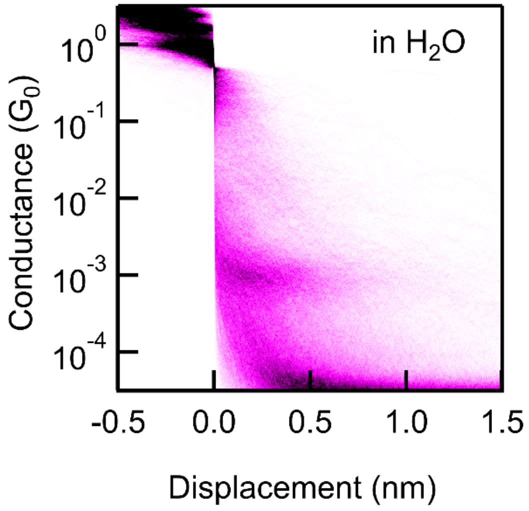

Figure S26. 2D conductance histogram of **Sti-NH$_2$** measured in H_2O under a 100 mV tip bias voltage.

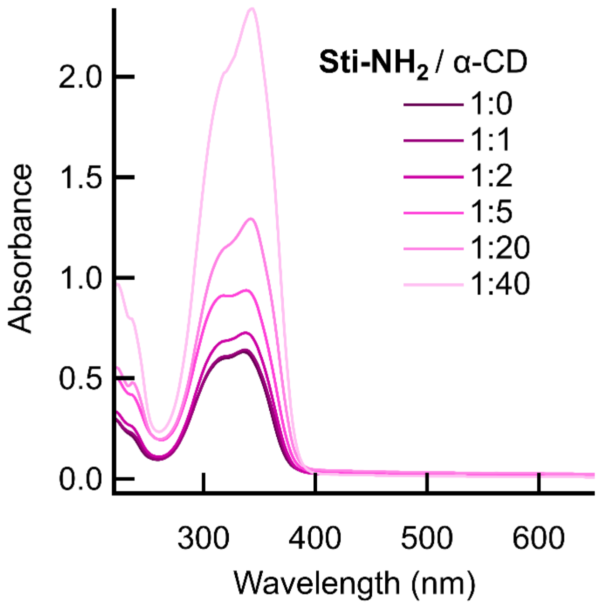

Figure S27. The UV-vis absorption spectra of **Sti-NH2** upon stepwise addition of excess α-CD. The concentration of **Sti-NH2** is kept at 1×10^{-4} M.

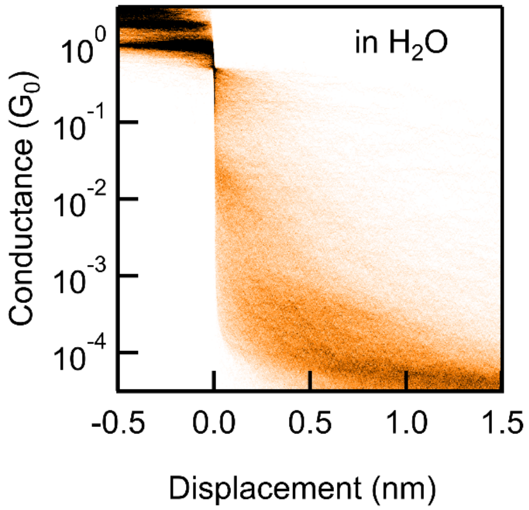

Figure S28. 2D conductance histogram of **Azo-SMe** measured in H_2O under a 100 mV tip bias voltage.

V. References

(1) Guo, W.; Quainoo, T.; Liu, Z.-F.; Li, H. Robust binding between secondary amines and Au electrodes. *Chem. Commun. (Cambridge, U. K.)* **2024**, *60* (25), 3393-3396. DOI: 10.1039/d3cc04284g.
(2) Jansook, P.; Ogawa, N.; Loftsson, T. Cyclodextrins: structure, physicochemical properties and pharmaceutical applications. *Int. J. Pharm. (Amsterdam, Neth.)* **2018**, *535* (1-2), 272-284. DOI: 10.1016/j.ijpharm.2017.11.018.
(3) Hernandez-Pascacio, J.; Pineiro, A.; Ruso, J. M.; Hassan, N.; Campbell, R. A.; Campos-Teran, J.; Costas, M. Complex Behavior of Aqueous α-Cyclodextrin Solutions. Interfacial Morphologies Resulting from Bulk Aggregation. *Langmuir* **2016**, *32* (26), 6682-6690. DOI: 10.1021/acs.langmuir.6b01646.
(4) Adak, O.; Rosenthal, E.; Meisner, J.; Andrade, E. F.; Pasupathy, A. N.; Nuckolls, C.; Hybertsen, M. S.; Venkataraman, L. Flicker Noise as a Probe of Electronic Interaction at Metal-Single Molecule Interfaces. *Nano Lett.* **2015**, *15* (6), 4143-4149. DOI: 10.1021/acs.nanolett.5b01270.
(5) Becke, A. D. Density-functional exchange-energy approximation with correct asymptotic behavior. *Phys. Rev. A: Gen. Phys.* **1988**, *38* (6), 3098. DOI: 10.1103/physreva.38.3098.
(6) Weigend, F.; Ahlrichs, R. Balanced basis sets of split valence, triple zeta valence and quadruple zeta valence quality for H to Rn: Design and assessment of accuracy. *Phys. Chem. Chem. Phys.* **2005**, *7* (18), 3297-3305. DOI: 10.1039/b508541a.
(7) Marenich, A. V.; Cramer, C. J.; Truhlar, D. G. Universal Solvation Model Based on Solute Electron Density and on a Continuum Model of the Solvent Defined by the Bulk Dielectric Constant and Atomic Surface Tensions. *J. Phys. Chem. B* **2009**, *113* (18), 6378-6396. DOI: 10.1021/jp810292n.
(8) Grimme, S.; Antony, J.; Ehrlich, S.; Krieg, H. A consistent and accurate ab initio parametrization of density functional dispersion correction (DFT-D) for the 94 elements H-Pu. *J. Chem. Phys.* **2010**, *132* (15), 154104/154101. DOI: 10.1063/1.3382344.
(9) Becke, A. D. Density-functional thermochemistry. III. The role of exact exchange. *J. Chem. Phys.* **1993**, *98* (7), 5648. DOI: 10.1063/1.464913.
(10) Lee, C.; Yang, W.; Parr, R. G. Development of the Colle-Salvetti correlation-energy formula into a functional of the electron density. *Phys. Rev. B: Condens. Matter* **1988**, *37* (2), 785. DOI: 10.1103/physrevb.37.785.
(11) Neese, F. The ORCA program system. *Wiley Interdiscip. Rev.: Comput. Mol. Sci.* **2012**, *2* (1), 73-78. DOI: 10.1002/wcms.81.
(12) Fishman, J. H.; Yarish, M. Gold-palladium electrocatalysts. *Electrochimica Acta* **1967**, *12* (5), 579-581. DOI: https://doi.org/10.1016/0013-4686(67)80028-8.
(13) Zang, Y.; Pinkard, A.; Liu, Z.-F.; Neaton, J. B.; Steigerwald, M. L.; Roy, X.; Venkataraman, L. Electronically transparent Au-N bonds for molecular junctions. *J. Am. Chem. Soc.* **2017**, *139* (42), 14845-14848. DOI: 10.1021/jacs.7b08370.
(14) Herrer, I. L.; Ismael, A. K.; Milan, D. C.; Vezzoli, A.; Martin, S.; Gonzalez-Orive, A.; Grace, I.; Lambert, C.; Serrano, J. L.; Nichols, R. J.; Cea, P. Unconventional single-molecule conductance behavior for a new heterocyclic anchoring group: pyrazolyl. *J. Phys. Chem. Lett.* **2018**, *9* (18), 5364-5372. DOI: 10.1021/acs.jpclett.8b02051.
(15) Deffner, M.; Gross, L.; Steenbock, T.; Voigt, B. A.; Solomon, G. C.; Herrmann, C. ARTAIOS - a transport code for postprocessing quantum chemical electro nic structure

calculations, available from https://www.chemie.uni-hamburg.de/ac/herrmann/software/index.html.
(16) Herrmann, C.; Solomon, G. C.; Subotnik, J. E.; Mujica, V.; Ratner, M. A. Ghost transmission: How large basis sets can make electron transport calculations worse. *J. Chem. Phys.* **2010**, *132* (2), 024103/024101. DOI: 10.1063/1.3283062.
(17) Papaconstantopoulos, D. A. *Handbook of the Band Structure of Elemental Solids*; Springer US, 1986.
